# DAXX-inducing phytoestrogens inhibit ER+ tumor initiating cells and delay tumor development

**DOI:** 10.1038/s41523-020-00178-5

**Published:** 2020-08-14

**Authors:** Daniel S. Peiffer, Emily Ma, Debra Wyatt, Kathy S. Albain, Clodia Osipo

**Affiliations:** 1 MD/PhD and Integrated Cell Biology Programs, Loyola University Chicago Stritch School of Medicine, Maywood, IL United States; 2 Department of Cancer Biology, Loyola University Chicago, Maywood, IL United States; 3 Department of Medicine, Division of Hematology/Oncology, Loyola University Chicago Stritch School of Medicine, Cardinal Bernardin Cancer Center, Maywood, IL United States; 4 Department of Microbiology and Immunology, Loyola University Chicago, Maywood, IL United States

**Keywords:** Breast cancer, Cancer stem cells

## Abstract

Recurrence of estrogen receptor (ER)-positive breast tumors despite curative-intent adjuvant therapy is thought to be due to enrichment of tumor initiating cells (TIC) during endocrine therapy (ET). Recently, it was identified that by antagonizing the ER, ET promotes rapid degradation of the death-associated factor 6 (DAXX) protein, which is necessary and sufficient to potently inhibit TICs. Thus, the goal of the current study was to identify a DAXX-inducing agent to inhibit TICs and prevent proliferation of the tumor. Phytoestrogens (naringenin, resveratrol, genistein, apigenin, and quercetin) were screened for DAXX protein expression, anti-TIC and anti-proliferative efficacy in vitro and in vivo. Specific DAXX-inducing phytoestrogens were tested to assess selectivity towards ERα and/or ERβ. Results showed that phytoestrogens tested induced DAXX protein expression and inhibited survival of TICs from ER+ MCF-7 and T47D cells. Only naringenin, resveratrol, and quercetin did not stimulate total cell proliferation. Naringenin, resveratrol, but not quercetin inhibited survival of TICs in vitro and in vivo in a DAXX-dependent manner. Naringenin-induced DAXX protein expression and inhibition of TICs seemed to be more selective towards ERβ while resveratrol was more selective through ERα. Naringenin or resveratrol inhibited the rate of tumor initiation and rate of tumor growth in a DAXX-dependent manner. These results suggest that a therapeutic approach using a phytoestrogen to induce DAXX protein expression could potently inhibit TICs within a tumor to delay or prevent tumor initiation. Therefore, a DAXX-promoting phytoestrogen should be explored for prevention of tumor progression in advanced disease and relapse in the adjuvant setting.

## Introduction

Cancer stem cells or tumor initiating cells (TICs) are a small population of cells within the breast tumor that may be a primary cause of cancer recurrence^1,2^. This is thought to be due to their stem-like properties, including their ability to self-renew and give rise to a heterogeneous tumor, ultimately contributing to poor clinical prognosis^3^. One pathway of clinical significance that promotes the survival of TICs is the NOTCH signaling pathway^4^. Specifically, NOTCH4 is required for the survival and self-renewal of TICs in estrogen receptor-positive (ER+) breast cancer^5–7^. Furthermore, NOTCH4 is activated in response to endocrine therapy (ET) to promote survival of TICs^5–8^. These findings suggest that in a subset of ER+ breast cancer, a proportion of TICs within a tumor may be resistant to ET and thus persist to promote recurrence of the cancer. This is reflected in clinical data illustrating high expression of a TIC marker, ALDH1, is associated with poor prognosis and higher likelihood of recurrence following ET^3^. Additionally, Pan et al. demonstrated that in patients with ER+ breast cancer given ET for 5 years, distant recurrence free survival rates during ET and the 5 years following therapy were almost identical^9^. This also suggests that ET alone may not be sufficient to prevent recurrence after standard, curative intent therapy in high risk patients and thus there is a clinical need for better therapeutics that prevent survival of TICs with the goal of preventing recurrence.

As TICs are dependent on NOTCH signaling for survival, and the canonical activation of NOTCH is already well described^10^, NOTCH inhibitors have been designed and evaluated for efficacy in ER+ breast cancer. One class of inhibitors that target NOTCH activation are gamma (γ)-secretase inhibitors (GSIs)^11^. GSIs prevent cleavage of the membrane-bound NOTCH receptor, thus preventing formation of the NOTCH intracellular domain (NICD)^12^. Inhibition of this final step in NOTCH activation prevents NICD from entering the nucleus to regulate gene expression required for TIC-survival^12^. The use of GSIs in combination with ET has been shown to restrict TIC-survival and growth of the tumor cell population, resulting in decreased tumor growth in preclinical models^6,13^. However, GSIs have yet to be clinically approved primarily due to gastrointestinal toxicity and increased risk of skin cancer in human patients^10^. Ultimately, there remains a clinical need for improved therapeutics that inhibit TIC survival without the associated toxicity.

We discovered that death domain-associated protein 6 (DAXX) is both necessary and sufficient to restrict NOTCH signaling, TIC survival in vitro and TIC frequency in vivo^14^. Furthermore, we demonstrated that the stability of the DAXX protein is dependent on 17β-estradiol (E_2_)-mediated ER activation^14^. Thus, ET-mediated inhibition of ER results in depletion of DAXX, activation of NOTCH4, and increased survival of TICs. This potentially presents a paradox where E_2_ activates ER to promote growth of the tumor, but yet suppresses TICs. However, use of the full agonist E_2_ is not a viable, therapeutic option as it promotes tumor growth. However, partial ER agonists could be beneficial if one is identified that does not stimulate tumor growth, but yet increases DAXX protein expression and potently inhibit TICs, thus potentially preventing tumor relapse^15–17^. Some partial agonists include natural estrogenic compounds called phytoestrogens found in citrus and plants^16,17^. Additionally, some phytoestrogens have been reported to be more selective for ERβ rather than ERα^18–20^. The hypothesis being that phytoestrogens may be sufficient to increase DAXX protein levels in an ERα/β-dependent manner without inducing total breast tumor cell proliferation. We tested the effects of a variety of phytoestrogens (naringenin, genistein, apigenin, resveratrol, and quercetin) on total cell proliferation of ER+ MCF-7 (wild-type p53) and T47D (mutant p53) cells, DAXX protein expression, and TIC survival in vitro and in vivo. We also tested if phytoestrogens may be more selective toward ERα or ERβ by using selective agonists or antagonists in vitro.

## Results

### Phytoestrogens induce DAXX without stimulating proliferation

**Fig. 1:**
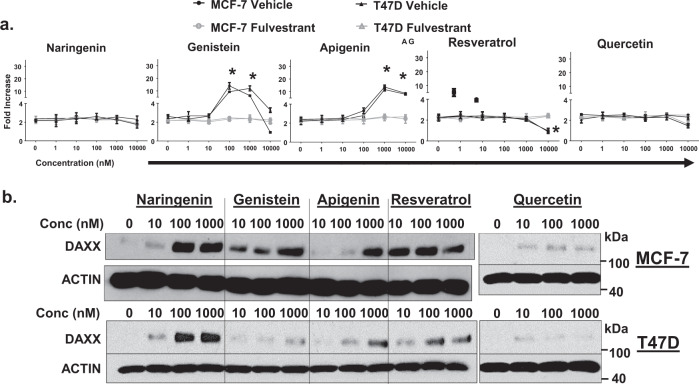
Phytoestrogens induce DAXX protein without stimulating proliferation. **a** MCF-7 and T47D cells were treated everyday with increasing concentrations of naringenin, genistein, apigenin, resveratrol, or quercetin from 0 to 10,000 nM for 7 days in charcoal-stripped FBS-containing medium. Ethanol was used as the vehicle and 100 nM fulvestrant was used as a pure ER antagonist. Fold increase in live cells was calculated as # of live cells at day 7/# of cells seeded at day 0. The graph depicts the mean ± the standard deviation (s.d.) of three replicates. Statistical significance between ethanol and the phytoestrogen treatment group was assessed using a Student’s *t*-Test. Asterisk denotes a *P* < 0.01. **b** Cells were lysed on day 7 and protein extracted for western blotting to detect DAXX and Actin proteins. Images are representative of three replicate studies.

Both ER+ MCF-7 and T47D cells proliferate in a concentration-dependent manner (0, 0.50, or 5.0 nM) in response to 17β-estradiol (E_2_) except at 50 nM, which is less stimulatory (Supplementary Fig. 1a). In contrast, as little as 0.50 nM E_2_ is sufficient to restrict TIC survival in vitro (Supplementary Fig. 1b). These data suggest that minimal activation of the ER may be sufficient to potently inhibit TICs but not stimulate proliferation of the majority of breast cancer cells. Phytoestrogens are known to activate ER signaling to varying degrees, some without inducing proliferation of ER+ breast cancer cells^16,17^. We screened five phytoestrogens (naringenin, genistein, apigenin, resveratrol, and quercetin) with distinct chemical structures for effects on total cell proliferation and DAXX protein expression using two ER+ breast cancer cells (MCF-7 and T47D). Genistein or apigenin stimulated total cell proliferation in a concentration-dependent manner ranging from 0 to 10,000 nM and this proliferation was dependent on ER as fulvestrant completely abrogated the effect (Fig. 1a). Conversely, neither naringenin, resveratrol, or quercetin stimulated proliferation up to 10,000 nM (Fig. 1a).

To determine if these phytoestrogens induced DAXX protein expression, western blotting was performed on lysates from MCF-7 and T47D cells treated with 0, 10, 100, and 1000 nM of each phytoestrogen for 72 h. Naringenin treatment at 100 nM increased DAXX protein compared to 0 nM in both MCF-7 and T47D cells (Fig. 1b). Apigenin increased DAXX protein expression at 1000 nM in both cell lines (Fig. 1b). Genistein or resveratrol increased DAXX at 10 nM but mainly in MCF-7 cells while quercetin only modestly increased DAXX in both cells (Fig. 1b). To assess if the phytoestrogen-mediated increase in DAXX stimulated classical ER signaling, expression of *TFF1* (PS2) transcripts were measured in cells-expressing or depleted for DAXX. Neither naringenin, resveratrol, or quercetin induced *TFF1* (PS2) expression to the same level as E_2_ (Supplementary Fig. 2). The modest increase in PS2 transcripts was not dependent on DAXX expression (Supplementary Fig. 2).

These findings indicate that phytoestrogens, naringenin and resveratrol, are sufficient to increase DAXX protein in ER+ breast cancer cells without stimulating total tumor cell proliferation or significantly activating classical ER signaling.

### ER or DAXX are required for inhibition of TIC survival by phytoestrogens

**Fig. 2:**
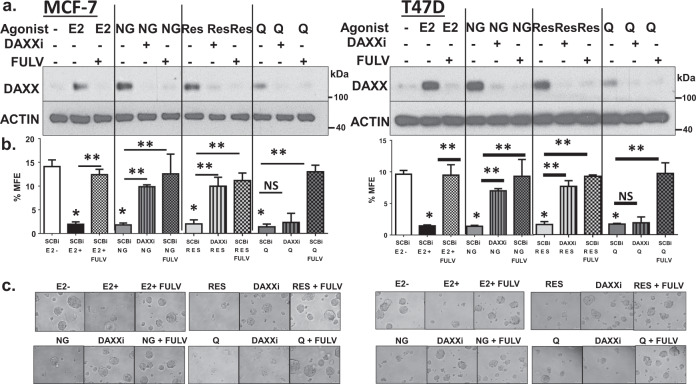
ER or DAXX are required for inhibition of TIC survival by phytoestrogens. **a** MCF-7 and T47D cells were transfected with a control (SCBi) or DAXX (DAXXi) siRNA for 48 h followed by treatment with a vehicle (ethanol), 5 nM E_2_, E_2_ + 100 nM fulvestrant (FULV), 100 nM naringenin (NG), 100 nM resveratrol (RES), or 100 nM quercetin (Q) alone or plus fulvestrant for 3 days. Western blotting was performed to detect DAXX and β-Actin proteins. Images are representative of three independent studies. **b** Cells were then plated onto low-attachment, six-well plates containing methylcellulose, mammosphere forming medium at a density of 50,000 cells/well. Plates were incubated at 37 °C for 7 days. Percent mammosphere forming efficiency was calculated based on the # of mammospheres counted/# of cells seeded × 100. The bar graphs are the mean ± s.d. of three independent studies. Statistical significance between groups was analyzed using a One-way ANOVA. The asterisk denotes significance between E2, NG, RES, or Q and the −E2 group under control siRNA (SCBi) conditions (*P* < 0.0001 for +E2, NG, RES, or Q). The double asterisk denotes significance between DAXXi and the SCBi group (*P* < 0.01 for NG, *P* < 0.001 for RES) or significance between FULV and +E2, NG, RES, or Q under control siRNA (SCBi) conditions (*P* < 0.0001 for +E2, *P* < 0.01 for the NG or RES, *P* < 0001 for Q). **c** Representative images of mammospheres taken at ×20 magnification. Scale bar = 100 μm.

To determine if naringenin or resveratrol inhibited survival of breast TICs through a DAXX-dependent manner, mammosphere forming efficiency (MFE) was performed on MCF-7 and T47D cells-expressing or depleted for DAXX. Expression of DAXX protein was detected by western blotting to confirm siRNA-mediated knockdown (Fig. 2a). Results showed that E_2_, naringenin, or resveratrol increased DAXX protein expression compared to the vehicle control (Fig. 2a). Quercetin also increased DAXX protein but to a lesser degree than E_2_, naringenin, or resveratrol. The increased DAXX protein by E_2_, naringenin, resveratrol, or quercetin was almost completely abrogated by fulvestrant (Fig. 2a), suggesting that the ER is required for DAXX protein expression. Results from MFE demonstrated that naringenin, resveratrol, or quercetin reduced survival of TICs similarly to E_2_ when compared to the vehicle control (Fig. 2b, c). Further, DAXX or the ER was required for the reduction in TIC survival as DAXX depletion by siRNA or inhibiting ER function using fulvestrant, respectively, rescued the decreased %MFE in response to E_2_, naringenin, or resveratrol (Fig. 2b, c). In contrast, quercetin-mediated decrease in TIC survival was dependent on the ER but not on DAXX expression (Fig. 2b, c). Together these findings indicate that phytoestrogens naringenin and resveratrol are sufficient to restrict TIC survival in an ER and DAXX-dependent manner. However, other phytoestrogens such as quercetin are potent inhibitors of TICs, but in a DAXX-independent manner.

The selective estrogen receptor downregulator (SERD) fulvestrant completely abrogated E_2_ or phytoestrogen-mediated inhibition of breast TICs in vitro, suggesting that the ER was required for TIC inhibition. To assess if the selective estrogen receptor modulator (SERM) tamoxifen had similar effects, MFE was measured from ER+ MCF-7 and T47D cells treated with a vehicle control (estrogen depletion), E_2_, E_2_+ 4-hydroxytamoxifen (4OHT), naringenin, naringenin + 4OHT, resveratrol, resveratrol + 4OHT, or quercetin, or quercetin + 4OHT. Treatment with 4OHT moderately prevented E_2_ or naringenin-mediated increase in DAXX protein in MCF-7 cells, while almost completely in T47D cells (Supplementary Fig. 3a). Interestingly, 4OHT was more effective at preventing resveratrol or quercetin-mediated increase in DAXX protein in both cell lines (Supplementary Fig. 3a). While fulvestrant almost completely abrogated E_2_, naringenin, resveratrol, or quercetin-mediated reduction of TICs from MCF-7 and T47D cells (Fig. 2b, c), 4OHT only restored TICs by 70–80% from MCF-7 and almost completely from T47D cells (Supplementary Fig. 3b). These results suggest the use of a phytoestrogen to inhibit breast TICs is probably most effective when used under estrogen deprivation therapy such as when combined with an aromatase inhibitor.

### Selectivity of phytoestrogens toward ERα, ERβ, or both to inhibit TICs

**Fig. 3:**
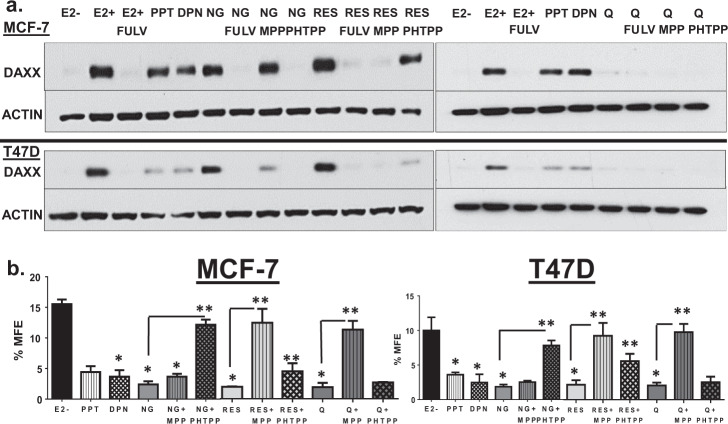
Phytoestrogens are more selective toward ERα or ERβ to induce DAXX and inhibit TICs. **a** MCF-7 (upper panels) and T47D (lower panels) cells were treated every day for 3 days with a vehicle (ethanol), 5 nM E_2_, E_2_ + 100 nM fulvestrant (FULV), 100 nM PPT (ERα agonist), 100 nM DPN (ERβ agonist), 100 nM naringenin (NG), 100 nM resveratrol (RES), 100 nM quercetin (Q), NG + FULV, NG + 100 nM MPP (ERα antagonist), NG + 100 nM PHTPP (ERβ antagonist), RES + FULV, RES + MPP, RES + PHTPP, Q + FULV, Q + MPP, or Q + PHTPP. All agents were diluted in charcoal-stripped FBS containing medium. Cells were lysed and DAXX and ACTIN proteins were detected by western blotting. Images are representative of three independent studies. **b** After treatments, cells were plated at a density of 50,000 cells/well into low-attachment six-well plates containing methylcellulose based mammosphere forming media and incubated for 7 days. Percent mammosphere forming efficiency was calculated based on the # of mammospheres counted/# of cells seeded × 100. Bar graphs show mean ± s.d. of three independent studies. Statistical significance was analyzed using a one-way ANOVA. The asterisk denotes significance between PPT, DPN, NG, RES, Q, and the −E2 treatment group (*P* < 0.0001). The double asterisk denotes significance between NG and NG + PHTPP, RES and RES + MPP, or Q and Q + MPP (*P* < 0.0001).

Previous reports have shown that naringenin, resveratrol, and quercetin may selectively activate specific ER isoforms^21^. Additionally, it has been demonstrated previously that expression of ERα is higher in bulk tumors compared to TICs-isolated from bulk tumors. Further, these reports showed that while ERα expression is low in TICs, ERβ expression is increased^22,23^. A pharmacologic approach was taken to determine if the phytoestrogen-mediated increase in DAXX protein and inhibition of TIC survival is through selective activation of ERα, ERβ, or both. The selective ERα agonist, PPT, or the ERβ agonist DPN induced similar levels of the DAXX protein, but to a lesser extent than E_2_, naringenin, or resveratrol alone (Fig. 3a) suggesting that both ER isoforms may be required for increased DAXX protein expression. The selective ERβ antagonist, PHTPP prevented naringenin-induced DAXX expression, while the selective ERα antagonist, MPP inhibited the resveratrol-induced DAXX expression (Fig. 3a). However, these ER antagonists are approximately 40X more selective for one isoform over the other, suggesting that DAXX protein expression is inducible by ERα, ERβ, or possibly both depending on the expression profile of each isoform. However, quercetin is a very weak inducer of DAXX possibly due to weak ER activity (Fig. 3a).

To assess the role of ERα or ERβ on TIC survival, MFE was performed on ER+ MCF-7 and T47D cells in response to a selective a ERα or ERβ agonist, phytoestrogens alone, and in combination with a selective ERα or ERβ antagonist. Either the ERα (PPT) or ERβ (DPN) agonist inhibited MFE (Fig. 3b). Naringenin-mediated inhibition of MFE was reversed by PHTPP, while resveratrol or quercetin-mediated reduction in MFE was rescued by MPP (Fig. 3b). These results suggest that activity of ERα, ERβ, or both receptors may be required for increased DAXX protein expression and inhibition of TIC survival by a phytoestrogen depending on the expression level of each ER isoform.

### Phytoestrogens repress NOTCH4 and NOTCH signaling in a DAXX-dependent manner

**Fig. 4:**
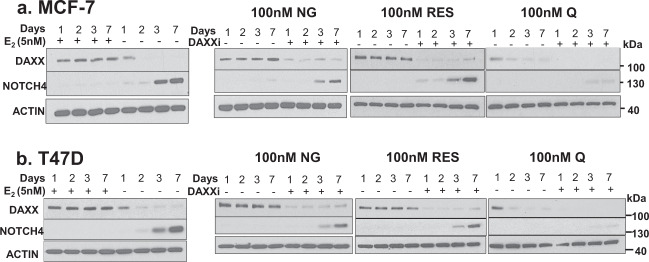
Phytoestrogens induce DAXX and repress NOTCH4. MCF-7 (**a**) or T47D (**b**) cells were transfected with a non-specific (SCBi) or DAXX-specific (DAXXi) siRNA for 2 days. Cells were grown in vehicle (ethanol), 5 nM E2 or 100 nM naringenin (NG), resveratrol (RES), or quercetin (Q) growth conditions. After 1–7 days, total cell lysates were collected and protein levels of DAXX, NOTCH4 and ACTIN were detected by western blot analysis. Images are representative of three independent studies.

**Fig. 5:**
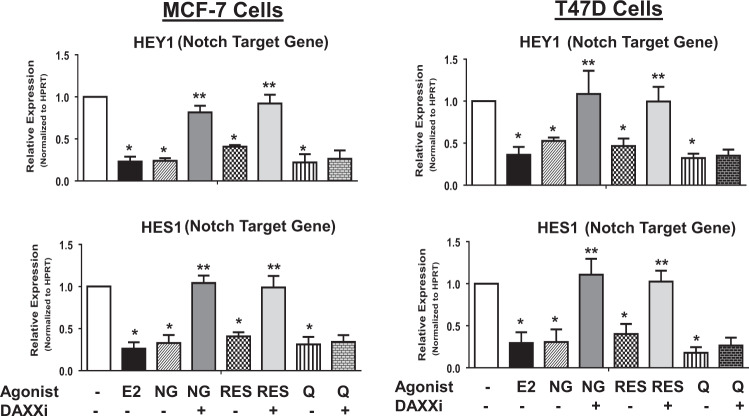
Phytoestrogens inhibit canonical Notch signaling. MCF-7 or T47D cells were treated as described in Fig. 3a. At 3 days, total RNA was isolated and reverse transcribed to cDNA. Real-time PCR was used to detect transcript levels of NOTCH targets HES1 and HEY1. Bar graphs show mean values ± s.d. of three independent studies of relative transcript expression normalized to HPRT and compared to SCBi + 0 nM E2 conditions from three independent experiments using the 2^−ΔΔCt^ calculation. A one-way ANOVA was performed on ΔCt values after initial normalization to HPRT. Symbols denote statistical significance of *p* < 0.01 between 5 nM E2, NG, RES, or Q vs. vehicle (*) or controli vs DAXXi (**).

Recently, it was shown that E_2_-mediated increase in DAXX protein potently inhibits TICs and NOTCH signaling^14^. To assess if phytoestrogens repress NOTCH4 and canonical gene targets (*HES1* and *HEY1)* through a DAXX-dependent mechanism, ER+ MCF-7 and T47D cells-expressing or depleted for DAXX were treated with naringenin, resveratrol, or quercetin and NOTCH4 protein and NOTCH target gene transcripts were measured. As previously shown, E_2_ is sufficient to maintain high DAXX protein levels from 1 to 7 days, while inhibiting the NOTCH4 protein (Fig. 4) and gene targets (Fig. 5). When cells are deprived of E_2_, DAXX expression decreases in a time-dependent manner to almost undetectable by day 2 (Fig. 4). NOTCH4 protein is detectable by day 2 or 3 and reaches a maximum by day 7 (Fig. 4) and gene targets are increased (Fig. 5). Similarly, naringenin or resveratrol maintain DAXX protein expression from 1–7 days while NOTCH4 protein is undetectable (Fig. 4) and gene targets are repressed (Fig. 5). Upon DAXX knockdown, NOTCH4 protein is detectable by day 3 and reaches a maximum by day 7 (Fig. 4) and gene targets are increased (Fig. 5). However, quercetin-mediated repression of NOTCH4 protein and gene targets does not seem to be dependent on DAXX (Figs. 4 and 5). These results indicate that DAXX is necessary for naringenin or resveratrol-mediated inhibition of NOTCH4 and NOTCH signaling.

### DAXX is necessary for phytoestrogen-mediated inhibition of tumor initiation

**Fig. 6:**
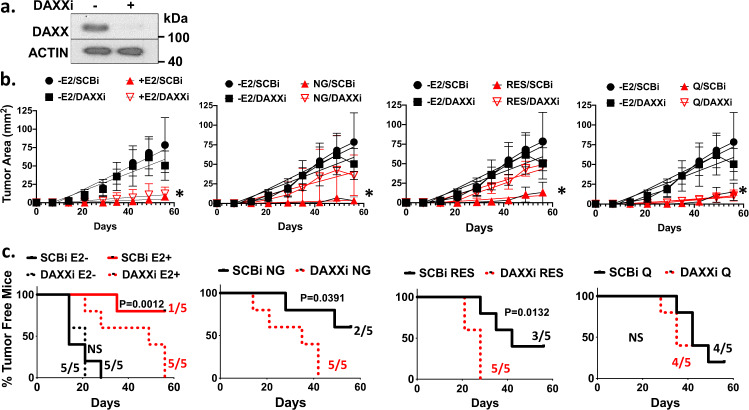
DAXX is necessary for phytoestrogen-mediated inhibition of tumor initiation. **a** MCF-7 cells were transfected with a control or DAXX siRNA for 48 h. Western blotting was performed to detect DAXX and Actin proteins. **b** Transfected cells were injected into mammary fat pads of female, ovariectomized, *foxn1* nu/nu, athymic nude mice. Each mouse was tagged on the ear with an identifiable number. Five mice received no estrogen capsule (E2−), five received an estrogen capsule (E2+), five were fed by oral gavage 20 mg/Kg naringenin (NG), 20 mg/Kg resveratrol (RES), or 20 mg/Kg quercetin (Q). Tumor area (mm^2^) was measured using Vernier calipers every week for 56 days (8 weeks). Graph shows mean ± s.d. of 1–5 tumors. Linear regression analysis was performed to calculate slopes for each tumor in a tagged mouse within a treatment group. A *t*-Test was used to assess statistical significance between slopes. The asterisk denotes a *P*-value < 0.0001 between slopes. **c** Rate of tumor initiation was determined by Kaplan–Meier analysis as percentage tumor free mice over 56 days. Statistical analysis was conducted using the Mantel-Cox Log rank test.

To assess if phytoestrogens inhibit tumor growth and/or tumor initiation in a DAXX-dependent manner, ER+ MCF-7-enriched TICs expressing or depleted for DAXX (Fig. 6a) were injected into female, athymic nude, ovariectomized mice to measure rates of tumor growth and tumor initiation when treated with E_2_ or a phytoestrogen. In the absence of E_2_, the growth rate of tumors and percentage tumor free mice were unaffected by DAXX depletion (Fig. 6b, c and Supplementary Fig. 4). E_2_ treated mice had small tumors and only 20% of the mice developed tumors (Fig. 6b, c, and Supplementary Fig. 4). DAXX depletion resulted in 100% of mice developing slightly larger tumors (Fig. 6b, c, and Supplementary Fig. 4), suggesting that E_2_-inducd DAXX expression inhibits TICs and tumor initiation. Naringenin or resveratrol-treated mice had slow tumor growth rates and only 40% and 60% of mice developed tumors, respectively (Figs. 6b, c, and Supplementary Fig. 4). DAXX depletion significantly increased the tumor growth rate and resulted in 100% of mice developing tumors (Fig. 6b, c, and Supplementary Fig. 4). DAXX expression had little effect on quercetin-treated mice in regards to tumor growth rate or percenatge of mice developing tumors (Fig. 6b, c, and Supplementary Fig. 4). These findings from MCF-7 cell line-derived xenograft studies in vivo suggest that a phytoestrogen (naringenin or resveratrol) increases DAXX to inhibit survival of breast TICs, restrict tumor growth rates and tumor initiation.

### The DAXX protein is stabilized by Naringenin

**Fig. 7:**
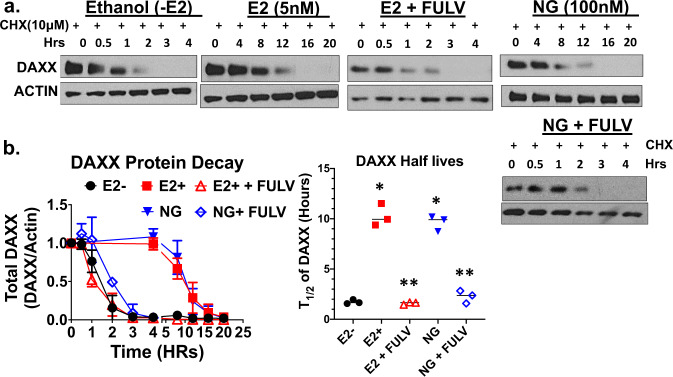
Naringenin increases the stability of the DAXX protein. MCF-7 cells at a density of 1 × 106 cells were grown in 0, 5 nM E2, E2 + 100 nM fulvestrant (FULV), 100 nM NG, or NG + fulvestrant (FULV) for 24 h, following which, 10 µM cyclohexamide (CHX) was added to the growth medium in the presence or absence of 10 µM MG132. Cells were incubated for their specified times (0–20 h) and Western blotting was conducted to detect DAXX and ACTIN proteins (**a**). The blot image is representative of three independent experiments. Densitometry analysis of each Western blot was performed using ImageJ software and the total DAXX protein density was plotted as a ratio of DAXX/ACTIN over a time in hours (**b** left graph) The half-life of DAXX for each experiment and condition was then determined by nonlinear regression analysis followed by one phase decay using GraphPad Prism 8 (**b** right graph). The determined half-life for each independent experiment was analyzed for statistically significant differences between the treatments using a one-way ANOVA. Asterisk denotes significance between E2 or NG and the E2− control treatment group (*P* < 0.05). Double asterisk denotes significance between E2+ FULV or NG + FULV and the E2 or NG treatment groups (*P* < 0.05).

The tumor initiating studies demonstrated that out of the three phytoestrogens tested, naringenin was the most potent at inhibiting the rate of tumor growth and tumor initiation in a DAXX- dependent manner. Thus, naringenin was selected to assess if it stabilizes the DAXX protein similar to estradiol using cycloheximide treatment as previously reported^14^. Results show that naringenin delays the decrease in DAXX protein expression similarly to estradiol when compared to the estrogen-deprived treatment (Fig. 7a). The half-life of the DAXX protein is increased by naringenin or estradiol to 10 h compared to ~2 h for the estrogen-deprived treatment (Fig. 7b). The increase in the half-life of the DAXX protein by naringenin or estradiol was dependent on the ER as fulvestrant abrogated both effects (Fig. 7b). These results suggest that at least naringenin stabilizes the DAXX protein possibly in a similar manner as estradiol.

### DAXX is necessary for naringenin-mediated repression of TIC-associated genes

**Fig. 8:**
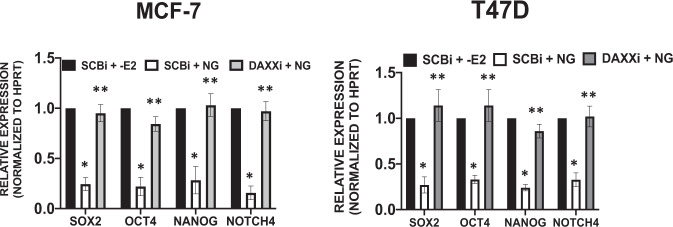
Naringenin inhibits expression of TIC genes in a DAXX-dependent manner. MCF-7 and T47D cells were transfected with a control (SCBi) or DAXX (DAXXi) siRNA for 48 h. Cells were grown in charcoal-stripped fetal bovine serum and treated with ethanol (−E2) or 100 nM naringenin (NG) for 24 h. Total RNA was extracted, reverse transcribed to cDNA, and real-time PCR was performed to detect SOX2, OCT4, NANOG, NOTCH4, and HPRT transcripts. Relative expression of each transcript was calculated using the 2^−ΔΔCt^ equation. The bar graph shows mean ± s.d. of three independent studies. Multiple *T*-Tests were performed on ΔCt values after initial normalization to HPRT to calculate statistical significance between treatment groups. The asterisk denotes statistical significance between the SCBi (−E2) and SCBi (NG) groups with *P*-values ≤ 0.0001. The double asterisk denotes statistical significance between the SCBi (NG) and DAXXi (NG) groups with *P*-values ≤ 0.002.

As the previous study demonstrated that naringenin stabilizes the DAXX protein similar to estradiol, naringenin was selected to assess if it also inhibited expression of TIC-associated genes and whether DAXX was necessary for this inhibition. The results from MCF-7 and T47D cells show that naringenin inhibited expression of SOX2, OCT4, NANOG, and NOTCH4 transcripts as compared to the estrogen-deprived group (Fig. 8). The decreased expression of these TIC-associated transcripts by naringenin was almost completely blocked by DAXX deletion (Fig. 8). These results suggest that naringenin requires DAXX to inhibit TICs by possibly repressing expression of TIC-associated genes.

### Phytoestrogens Induce DAXX Protein in ER+ PDX Tumors in vivo and Inhibit TICs ex vivo

**Fig. 9:**
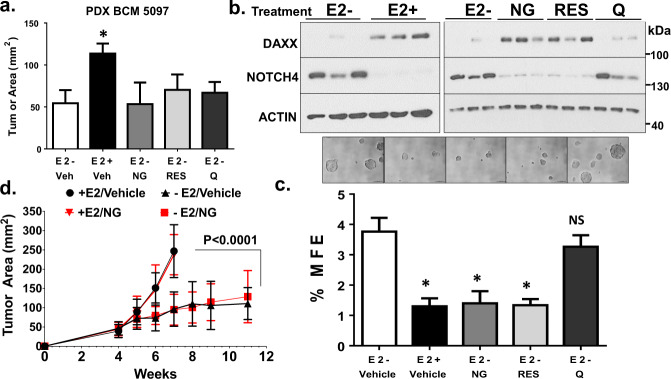
Phytoestrogens induce DAXX protein and inhibit TICs in vivo. **a** ER + BCM 5097 PDX tumor pieces at 0.1–0.2 mm in length were subcutaneously implanted into female, ovariectomized, *foxn1 nu/nu*, athymic nude mice. All Mice were implanted with a 0.30 cm silastic release capsule containing 17β-estradiol. Tumors were allowed to grow to a mean area of 9 mm^2^ and mice were randomized to the following treatments: 1. Vehicle (PBS), E_2_ capsule removed (E2−), 2. Vehicle (PBS), E_2_ capsule retained (E2+), 3. 20 mg/kg naringenin (NG), 4. 20 mg/kg resveratrol (RES), or 20 mg/kg quercetin (Q), E_2_ capsule removed (E2−) by oral gavage daily for 2 weeks. Tumor area (mm^2^) was measured using Vernier calipers at the end of the study. Bar graphs show mean ± s.d. of 4–5 tumors. A Student’s *t*-Test was used to calculate statistical significance between groups. The asterisk denotes statistical significance of *P* < 0.05 between the E2+ and E2− groups. **b** Tumors (*N* = 3) were extracted, minced, and treated with collagenase to isolate single cells. DAXX, NOTCH4, and β-Actin proteins were detected in three tumors per treatment by western blotting. **c** Freshly isolated cells at a density of 50,000 cells/well were plated onto low-attachment six-well plates containing methylcellulose based mammosphere forming media and incubated for 7 days. Percent mammosphere forming efficiency was calculated based on the # of mammospheres counted/# of cells seeded × 100. Bar graphs show mean ± s.d. of three replicates. The asterisk denotes statistical significance of *P* < 0.001 between E2+, NG, or RES and the E2- group as analyzed using a Student’s *t*-Test. Representative images were taken at ×20 magnification. Scale bar = 100μm. **d**vIn a separate study, ER + BCM 5097 PDX tumor pieces at 0.1–0.2 mm in length were subcutaneously implanted into 10 female, ovariectomized, *foxn1 nu/nu*, athymic nude mice. All Mice were tagged with a number on their ears and were implanted with a 0.30 cm silastic release capsule containing 17β-estradiol. Tumors were allowed to grow to a palpable mean area of 10 mm^2^ and mice were randomized to the following treatments: 1. Vehicle (PBS), E_2_ capsule removed (E2−), 2. Vehicle (PBS), E_2_ capsule retained (E_2_+), 3. 20 mg/kg naringenin, E_2_ capsule retained (E_2_+), and 4. 20 mg/kg naringenin, E_2_ capsule removed (E2−) by oral gavage daily for 11 weeks. Tumor area (mm^2^) was measured weekly using Vernier calipers. Graph shows mean ± s.d. of 9–10 tumors. Linear regression analysis was conducted to determine the slope of each tumor within a treatment group and statistical significance between slopes was assessed using a *t*-Test.

To determine effects of phytoestrogens, naringenin, resveratrol, or quercetin on initial tumor growth and TICs within the bulk tumor, an ER+ PDX tumor (BCM-5097) was implanted subcutaneously in female, athymic nude, ovariectomized mice and treated with a vehicle (no E_2_), E_2_, naringenin, resveratrol, or quercetin for 2 weeks. Only the E_2_ treated mice developed tumors exceeding 100 mm^2^ in area compared to the vehicle control (Fig. 9a). Neither of the phytoestrogens stimulated tumor growth above the vehicle control, at least up to 2 weeks (Fig. 9a). To assess effects on DAXX and NOTCH4 protein expression and TIC survival, the PDX tumors were excised at the 2-week time point, minced, and digested into single cell suspensions. Western blotting showed that naringenin or resveratrol increased DAXX protein expression in three tumors, while decreasing NOTCH4 protein expression, similar to the E_2_ treated group (Fig. 9b). Naringenin or resveratrol inhibited TIC survival as assessed by %MFE similar to E_2_ (Fig. 9c). Quercetin had little effect on DAXX and %MFE (Fig. 9c). These results show that naringenin or resveratrol restricts survival of TICs within a human ER+ PDX tumor.

The previous results showed that short-term use of phytoestrogens did not stimulate tumor growth. As most endocrine-based therapies are given long term, we investigated if longer use of a phytoestrogen (naringenin) would stimulate tumor growth of the ER+ PDX tumor. E_2_ alone or in combination with naringenin rapidly stimulated tumor growth reaching a mean of 250 mm^2^ in area within 6 weeks (Fig. 9d). Estrogen deprivation or naringenin alone increased tumor growth at similar rates reaching nearly 100 mm^2^ in area within 11 weeks (Fig. 9d). This result from a longer in vivo study suggests that at least naringenin does not stimulate tumor growth compared to estrogen deprivation.

### Summary of results

**Fig. 10:**
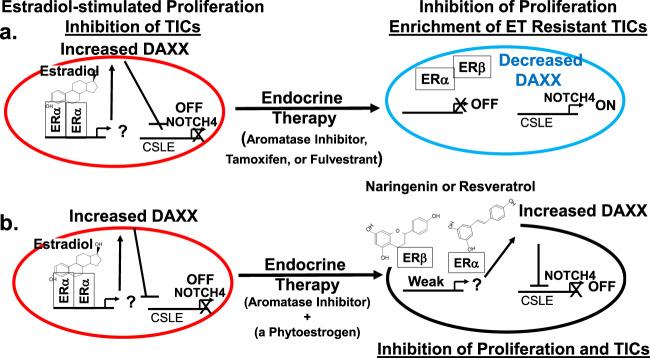
Summary of results. **a** Estradiol-mediated ERα transcription stimulates proliferation of breast cancer cells by regulation of classical estrogen responsive elements (ERE) and/or non-classical transcriptional factor responsive elements including AP-1, Sp1, or CMYC. Estradiol increases DAXX protein expression through an unknown mechanism. High DAXX protein represses Notch-dependent CSL elements (CSLE) and thus NOTCH4 resulting in inhibition of TICs. The unintended consequence of targeting ER using endocrine therapy (estrogen deprivation, tamoxifen, or fulvestrant) is decreased DAXX protein, de-repression of NOTCH4 and enrichment of ET resistant TICs. **b** A phytoestrogen including naringenin or resveratrol is a weak ERβ and/or ERα agonist but a potent DAXX protein inducer and inhibitor of NOTCH4 and breast TICs without the adverse effect of stimulating proliferation. The results herein suggest that a combination of a phytoestrogen or other new DAXX-promoting agents in combination with an aromatase inhibitor may inhibit both total proliferation and TICs.

These studies demonstrate that estrogenic agents have potential beneficial effects. Estradiol, while it stimulates proliferation of ER+ breast cancer cells, is a potent stabilizer of the DAXX protein and repressor of NOTCH4 and other stem cell genes thus inhibiting TICs. The unintended consequence of targeting ER using ET is degradation of DAXX, de-repression of NOTCH4 and other stem cell genes and enrichment of ET resistant TICs. The current study shows that a phytoestrogen including naringenin or resveratrol is also a potent inducer of the DAXX protein and potent inhibitor of breast TICs possibly through selective activation of ERα or ERβ depending on expression levels. Importantly, neither naringenin nor resveratrol had the adverse effect of stimulating bulk cell proliferation or tumor growth. The results herein suggest that a combination of a phytoestrogen or other new DAXX-promoting agents in combination with an aromatase inhibitor may inhibit both total proliferation and TICs (Fig. 10). Thus, DAXX could be used as a new biomarker to determine efficacy of these phytoestrogens and possibly other new DAXX-promoting agents on tumor development and recurrence.

## Discussion

We discovered in this study that a phytoestrogen, either naringenin or resveratrol, potently inhibits breast TICs in vitro and in vivo without stimulating proliferation of tumor cells specifically by increasing the DAXX protein. At least one phytoestrogen, naringenin stabilized the DAXX protein in an ER-dependent manner similarly to estradiol. The implication of this study is that tumor recurrence of ER+ breast cancer could possibly be prevented by using a phytoestrogen or a DAXX-stabilizing agent. Results from this current study showed that naringenin or resveratrol-induced DAXX protein expression and potently inhibited breast TICs when given in the absence of 17β-estradiol, and not when combined with 4OHT or fulvestrant. Thus, the best possible therapeutic approach for eliminating TICs and possibly eradicating ER+ breast cancer could be combining a phytoestrogen or another DAXX-promoting agent with an aromatase inhibitor (summarized in Fig. 10).

Previously, it was demonstrated that DAXX protein expression requires activation of the ER^14^. In the current study, phytoestrogens seemed to increase DAXX and inhibit TICs selectively through ERα, ERβ, or both. Naringenin seemed to be more selective towards ERβ while resveratrol was more selective towards ERα to induce DAXX protein and restrict survival of TICs. Interestingly, quercetin was also a potent inhibitor of TICs without inducing high levels of DAXX. The differences between the phytoestrogens on DAXX expression and TIC inhibition could be due to activation of ER isoforms. Quercetin is a weak activator of classical ER signaling as assessed by induction of PS2 transcripts, while naringenin and resveratrol are slightly stronger inducers of PS2 transcripts (Supplementary Fig. 2). Future studies using RNA and ChIP sequencing will investigate if one or more phytoestrogens induce differential gene expression programs as compared to estradiol to induce DAXX expression and inhibit TICs.

These findings are supported by recent results from the Women’s Health Initiative (WHI) trials. Hormone replacement therapy (HRT) in the form of estrogen alone decreased incidence of invasive breast cancer by nearly 30% compared to placebo controls^24^. However, estrogen alone increased benign proliferative disease by more than two fold^25^. These results suggest that estrogen therapy could be promoting more differentiated, less harmful benign disease, but inhibiting more aggressive invasive disease. It is possible that estrogens may have beneficial effects by stimulating growth of a less heterogeneous tumor. Therefore, DAXX could potentially be used as a biomarker for detection of heterogeneity within a tumor and response to new and improved anti-TIC agents.

Previous reports on phytoestrogens showed beneficial effects on breast cancer risk. Population health studies demonstrated that relatively high dietary intake of phytoestrogen-containing foods is associated with lower risk of breast cancer. These benefits were lost when women migrated from countries rich in phytoestrogen-containing diets to countries low in phytoestrogen-containing diets^26^. Preclinical studies support these findings. Specifically, naringenin, resveratrol, or quercetin was shown to reduce carcinogen-induced breast tumorigenesis in vivo^27–29^.

The use of a phytoestrogen in the clinical trial setting needs additional pre-clinical validation. For example, phytoestrogens are readily metabolized, in part, by the liver cytochrome P450 enzymes, thus potentially decreasing bioavailability to the tumor^30,31^. Also, phytoestrogens alter estrogen synthesis and metabolism, thus potentially affecting response to ET^32^. The results from the current study provide a “proof of concept” that weak, partial ER agonists such as phytoestrogens could potentially be explored in future validation studies for prevention of tumor recurrence and/or progression of metastatic disease. This study provides a basis for the design of new and improved agents with weak estrogenic activity rather than complete ER blockade specifically for elimination of breast TICs and prevention of tumor relapse. This study also suggests that DAXX could be used as a biomarker of response to these types of agents.

## Methods

### Cell culture

MCF-7 and T47D cells were purchased from American Type Culture Collection (ATCC, Manassas, VA). Both cell lines were grown and maintained in Roswell Park Memorial Institute Medium (RPMI-1640, Thermo Fisher Scientific, Waltham, MA). RPMI-1640 was supplemented with 10% Fetal Bovine Serum (FBS, Gemini Bio Products, Sacramento, CA), 1% (2 mM) l-glutamine (Thermo Fisher Scientific, Waltham, MA) and 1% (100 µM) non-essential amino acids (Invitrogen, Carlsbad, CA). For experimental conditions, phenol red-Free RPMI-1640 (Thermo Fisher Scientific Waltham, MA) was used and supplemented with 10% dextran charcoal-stripped FBS to remove E_2_ from the solution as well as 2 mM l-glutamine and 1% non-essential amino acids. All cell lines were authenticated in December 2018 by short tandem repeat allelic profiling (ATCC, Manassas, VA) and maintained at a low passage number (20 or less passages/cell line) to avoid clonal selection. All cell lines were maintained in a 37 °C incubation chamber at 95% humidity and 5% CO_2_. Cell medium was changed every day.

### Patient-derived xenografts (PDXs)

All animal studies were approved by Loyola University Chicago’s Institutional Animal Care and Use Committee (IACUC). The PDX BCM-5097 was purchased from The Baylor College of Medicine^33^. This primary breast tumor was originally derived from a female Caucasian patient with metastatic disease following pre-surgical docetaxel treatment^33^. It is positive for both the ER and progesterone receptor (PR) and negative for overexpression of human epidermal growth receptor 2 (HER2). The tumor was originally passaged in female, ovariectomized NOD/SCID mice implanted with an E_2_-containing capsule allowing a constant release of E_2_ to mimic post-menopausal levels (83.8 pg/mL) for two total passages^34^. Tumors were then implanted in ovariectomized *FoxN1* nu/nu athymic nude mice implanted with an E_2_-containing capsule for 2 or 11 weeks, depending on the study. Capsules were then removed from E2− + vehicle and all PE-treated groups for ex vivo studies.

### Drugs and chemicals

E_2_ was purchased from Sigma Aldrich (Catalogue # E8875) and suspended in 100% ethanol to form a 5 µM stock solution which was protected from light and maintained in −20 °C. This solution was diluted in growth medium to form a working concentration of 5 nM. Fulvestrant, a selective ER down regulator (SERD), and tamoxifen, a selective ER modulator (SERM) were purchased from Selleck Chemicals (Houston, TX) and a 100 µM stock was made by suspending the compound in 100% ethanol to a stock concentration and maintained at −20 °C. A working solution of 100 nM was formed by diluting the stock solution in experimental medium. The Phytoestrogens, naringenin (NG, N5893-25G), quercetin (Q, Q4951-10G) and resveratrol (RES, R5010-500MG) were purchased from Sigma Aldrich. The Phytoestrogens, genistein (GS, S1342) and apigenin (AG, S2262) were obtained from Selleck Chemicals (Houston, TX). Lyophilized material was initially suspended in dimethylsulfoxide (DMSO) to a stock concentration of 100 mM and stored from light at −20 °C. Stock solutions were then diluted in growth medium to form the appropriate working concentration. ER-isoform selective agonists and antagonists were generously provided by Dr. Stephanie Watkins. They include the ERα-specific agonist 4, 4′, 4″-(4-Propyl-[1H]-pyrazole-1, 3, 5-triyl)trisphenol (PPT) and antagonist 1, 3-Bis(4-hydroxyphenyl)−4-methyl-5-[4-(2- piperidinylethoxy)phenol]-1H-pyrazole dihydrochloride (MPP)^35,36^. The ERβ-specific agonist and antagonist used are 2, 3-bis(4-Hydroxyphenyl)-propionitrile (DPN) and 4-[2-Phenyl-5, 7-bis(trifluoromethyl)pyrazolo[1, 5-a]pyrimidin-3-yl]phenol (PHTPP) respectively^37,38^.

### RNA interference and transfection reagents

A pool of four DAXX small interfering RNAs (siRNAs) were used to artificially knockdown DAXX expression. These sequences include DAXXi-A (CAGCCAAGCTCTATGTCTA), DAXXi-B (GGAGTTGGATCTCTCAGAA), DAXXi-C (GAGGTTAACAGGCGCATTG), DAXXi-D (GCAAAACAAAGGACGCATA), which were purchased from Dharmacon GE Life Sciences (Lafayette, CO). A non-targeting scrambled control siRNA (SCBi) was purchased from Qiagen (Germantown, MD). The transfection reagent Lipofectamine RNAiMAX (Catalogue # 13778150) was purchased from Thermo Fisher Scientific (Waltham, MA). Transfection conditions consisted of using a ratio of 1:1 with 10 nM of appropriate siRNA according to the manufacturer’s protocol. Cells were incubated in transfection medium for 2 days for all experiments.

### Cycloheximide DAXX protein decay

MCF-7 cells at a density of 1 × 106 cells were grown in 0, 5 nM E2, E2 + 100 nM fulvestrant, 100 nM NG, or NG + fulvestrant for 24 h, following which, cyclohexamide (CHX) was added to the growth medium to form a final concentration of 10 µM in the presence or absence of the proteasome inhibitor MG132 (10 µM). Cells were incubated for their specified times (0–20 hours) and total protein was isolated and processed to create 20 μg protein lysates as described in the Western Blot Analysis methods. The relative amounts of DAXX for each experimental condition were determined by running the lysates on an 8% Tris-glycine gel as described in the Western Blot Analysis methods. These experiments were conducted in triplicate. Densitometry analysis of the Western blot was done using ImageJ software and the total DAXX protein density was plotted as a ratio of DAXX/ACTIN. The half-life of DAXX for each CHX experiment and condition was then determined by nonlinear regression analysis followed by one phase decay using GraphPad Prism 8. The determined half-life for each independent experiment was analyzed for statistically significant differences between the treatments using a One-Way ANOVA.

### Western blot analysis

MCF-7 and T47D cells were treated under their respective conditions, following which cells were washed twice with ice-cold phosphate-buffered saline (PBS). 300μL of lysis buffer (1% TritonX-100, 50 mM HEPES, 150 mM sodium chloride, 5 mM ethylenediaminetetraacetic acid (EDTA), 1 mM phenylmethylsulfonyl fluoride (PMSF), 1 mM sodium orthovanadate, 10 mM sodium fluoride (NAF) and 1 protease inhibitor cocktail tablet (Thermo Fisher Scientific) was used to lyse cells. Cells were scraped on each plate and the fluid lysate was collected. The lysate was incubated on ice for 10 min, following which cells were sonicated three times for 10 s/each using the Sonic Dismembrator (Model 100, Thermo-Fisher Scientific, Waltham MA). PDX (BCM-5097) tumor samples were isolated in the ex vivo experiments, half of the resultant tumor was snap frozen in liquid nitrogen. Tumors were then individually crushed into a powder using a mortar and pestle, with the subsequent powder being resuspended in the lysis buffer outlined for the cellular lysate preparation. Lysates were then incubated on ice for 10 minutes, following which samples were sonicated five times, 10 s/each. Samples were clarified by spinning at 4 °C at 14,000 rpm for 10 min. The supernatant was isolated to determine protein concentration. Protein concentration of cellular and PDX lysates was determined using Pierce bicinchoninic acid (BCA Protein Assay Kit (Thermo Fisher Scientific, Waltham, MA, Cat # 23225). A 20 µg in 30–40 µL solution of western blot lysates were prepared using 2X Laemmli buffer (BioRad, Hercules, CA Catalogue # 1610737) and β-mercaptoethanol (Thermo Fisher Scientific, Waltham, MA, Catalogue # BP-176-100). Samples were boiled for 10 minutes at 95 °C to denature proteins. Proteins were separated by molecular weight using SDS-PAGE buffered with 8% tris-glycine. Relative molecular weight was determined by including the HiMark Prestained protein standard (Thermo Fisher Scientific, Waltham, MA, Catalogue # LC5699). Proteins were separated by running the gel for 60 min at 150 V for in Tris-glycine SDS Running Buffer. Separated proteins were transferred to a nitrocellulose membrane using 100 V for 90 min. Following this transfer, the protein-containing membrane was blocked using 5% non-fat dry milk (DAXX, β-ACTIN) or 20% Roche (NOTCH4) buffer for 60 minutes at room temperature. TBST (5 mM Tris-HCL, 5 mM Tris-base, 150 mM sodium chloride, 0.05% Tween-20 and 0.2% NP-40 at pH 8.0) was used for dilution of both the milk and Roche buffers. Following blocking, the primary antibodies DAXX (1:1000, clone 25C12, Rabbit anti-human, Cell Signaling Technology #4533), β-ACTIN (1:2000, clone AC-15, mouse anti-human, Sigma Aldrich #A5441), NOTCH4 (1:1000, clone A-12, mouse anti-human, Santa Cruz Biotechnologies #sc-393893) were diluted in 5% milk or 20% Roche and added to the membrane for overnight incubation at 4 °C with constant agitation. The membranes were then washed 3x in TBST for 10 minutes per wash. Appropriate HRP-conjugated secondary antibody (Cell Signaling Technologies) diluted in 5% milk was added to the membrane, incubated for 60 minutes at room temperature, and finally washed 3x in TBST for 10 minutes per wash. Proteins were detected and visualized using enhanced chemiluminescence (ECL) Western Blotting Substrate (Pierce, Rockford, IL) and exposing the membrane to X-ray film in a dark room. Re-probing was performed by washing the membrane in TBST and then membrane stripping buffer for 5 min incubations 2X. The membrane was then blocked in 5% milk or 20% Roche buffer and re-probed with the appropriate primary antibody. All western blots were derived from the same experiment and were processed in parallel.

### Real-time PCR

MCF-7 and T47D cells were exposed to specified growth conditions and total RNA was extracted according to the manufacturer’s protocol using the RiboPure RNA Purification Kit (Ambion, Austin, TX, Catalogue # AM1924) and RNA concentration was determined using a NanoDrop Spectrophotometer (Therm Fisher Scientific, Waltham, MA). Reverse transcription (RT) was performed on RNA to synthesize cDNA using a reverse transcriptase enzyme kit. Briefly, 1 µg of RNA was diluted into 50μL of reaction volume (1X Reverse Transcriptase Buffer, 5.5 mM MgCl_2_, 500 µM dNTPs, 2.5 µM random hexamers, 0.4U/µL RNase inhibitor and 1.25U/µL Reverse Transcriptase enzyme, Multiscribe^TM^ Reverse Transcriptase Kit, Applied Biosystems, Foster City, CA, Catalogue # N8080234). RT reaction conditions were 10 min at 25 °C, 30 min at 48 °C, 5 minutes at 95 °C, 60 min at 25 °C, and maintained at 4 °C until ready for use. Real-time PCR was carried out using iTaq^TM^ SYBR® Green Supermix (Biorad, Hercules, CA) in order to detect transcript levels of a control gene Hypoxanthine-guanine phosphoribosyltransferase (HPRT), SOX2, OCT4, NANOG, NOTCH4, and NOTCH target genes, HES1 and HEY1. The PCR reaction was performed at 10 minutes at 95 °C, 40 cycles of 10 s at 95 °C then 45 s at 60 °C. Following this, 40 cycles of a melt curve was conducted as a control as outlined by the manufacturer of the StepOnePlus Real-time PCR machine (Applied Biosystems, Foster City, CA). HPRT was used as a loading control allowing for normalization of C_t_ expression for each gene transcript and experimental group. Relative fold-change in transcript expression between each experimental sample was calculated using the 2^(−ΔΔCt)^ method as outlined: Δ*C*_t_ Experimental = (*C*_t_ value of experimental gene of experimental group-C_t_ value of HPRT of experimental group), Δ*C*_t_ control = (*C*_t_ value of experimental gene of control group − *C*_t_ value of HPRT of control group), ΔΔ*C*_t_ = (Δ*C*_t_ experimental group − Δ*C*_t_ control group), relative quantity (RQ) = 2^(−ΔΔCt)^. All primer sequences used for these reactions are listed in Supplemental Table 1.

### Total cell proliferation

MCF-7 or T47D cells at a density of 100,000 cells were plated in individual wells of a 6-well plate and allowed to adhere for 24 h. Cells were then washed with PBS 2X, and specified growth medium was added containing vehicle, 5 nM E_2_, or appropriate concentration of PE in the presence or absence of fulvestrant. The growth medium was changed daily for a total of 7 days, following which cells were trypsinized and total viable cells were counted using a Countess Cell Counter. Fold increase in live cell number was calculated by dividing the total viable cells at day 7 by the number of cells initially plated at day 0 (100,000 cells).

### Mammosphere forming assay

TIC-survival was assessed using the mammosphere forming assay in vitro. The protocol used was adapted from Shaw et al.^39^ and previously described in Peiffer et al.^14^. In brief, DMEM-F12 medium (Gibco, Catalogue # 11039021) was heated to 60 °C and 2 g of methylcellulose was added. The solution was continuously stirred at 60 °C for 2.5 h until the methylcellulose was completely dissolved. The medium was incubated overnight at 4 °C with constant stirring. The following day, 4 mL of B-27 supplement and 4 µL of recombinant human epidermal growth factor (hEGF, Sigma Aldrich, Catalogue # E-9644) were added and the solution was stirred for 30 min at 4 °C. The medium was then transferred to centrifuge tubes and centrifuged at 9500 rpm in a Beckman rotor for 30 min at 4 °C any precipitate from the solution. The solution (mammosphere medium) was transferred into 50 mL conical tubes and stored at −20 °C until use. MCF-7 and T47D cells were grown in the specified experimental conditions and trypsinized for counting. 50,000 cells/well were then deposited in the mammosphere medium and incubated for 7 days at 37 °C. Mammospheres were then visualized and counted using a light microscope at ×20 magnification and harvested. The Mammosphere Forming Efficiency (MFE) was calculated using the following equation: %MFE = [(total number of mammospheres counted) × (dilution factor)]/(50,000 cells) × 100.

### Ex vivo analysis of TIC-survival

The protocol for these animal studies was approved by Loyola University Chicago’s Institutional Animal Care and Use Committee (IACUC). Pieces of the PDX BCM-5097 tumor of ~2–3 mm in length were implanted into the mammary fat pad of female, ovariectomized *FoxN1* nu/nu athymic mice (Harlan Sprague-Dawley, Madison, WI). A 0.30-cm E_2_ containing capsule was simultaneously implanted with the PDX tumor. Tumors were allowed to propagate for 2 or 11 weeks, following which the capsule was removed in animals treated with E_2_− + vehicle and PE-treated groups. Animals were fed by oral gavage with either vehicle (100μL PBS) or a solution containing 20 mg/kg of NG, RES or Q. Animals were fed daily for 5 days on, 2 days off for 14 days for the short-term study or for the NG long term study (11 weeks). These concentrations of PE were used based on previous reports^27,28^. Tumor area was measured weekly using a vernier caliper. After 14 days, tumors from each animal were excised with ½ being snap frozen to quantify DAXX and NOTCH4 protein levels via Western blot analysis.

The remaining ½ of the tumor was digested into a single cell solution for ex vivo analysis of TIC-survival via the mammosphere assay. PDX tumors were minced into small pieces and resuspended in growth medium containing RPMI, 10% FBS, l-glutamine, non-essential amino acids and 1% penicillin/streptomycin (HyClone SV30010). Tumor bits were placed in a 50 mL tube and centrifuged at 1200 RPM for 5 mins. The supernatant was decanted and the pellet was washed in 5 mL of cold PBS 2x, with a 1200 rpm spin for 5 mins after each wash. The pellet was then resuspended in RPMI lacking FBS, l-glutamine, NEAA or antibiotics with the addition of 50 U/mL of dispase II (Sigma Aldrich, D4693) and 0.1% collagenase II (Gibco, 17018-029). The solution + PDX pellet was vortexed and incubated at 37 °C for 1 h, vortexing every 20 mins. The solution was vortexed a final time and run through an 85 μM filter. Remaining debris was squeezed to remove any residual cells. The filtered cells were resuspended in growth medium (RPMI + 10% FBS, +l-glut, + NEAA, +1% antibiotics). The pellet was centrigued at 1200 rpm for 5 mins. The supernatant was decanted and the pellet was resuspended in 5 mL of PBS. Cells were then counted via trypan blue staining and 50,000 cells/PDX tumor were added to individual wells containing 3 mL of mammosphere medium. Plates were incubated at 37 °C for 7 days and %MFE was determined as described above.

### Tumor initiating potential in vivo

The protocol for these animal studies was approved by Loyola University Chicago’s Institutional Animal Care and Use Committee (IACUC). MCF-7 cells were transfected with SCBi or DAXXi siRNA for 2 days, following which cells were plated in mammosphere medium at a concentration of 50,000 cells/well. Cells were allowed to propagate for 7 days at 37 °C. Mammospheres were then isolated as described above and resuspended in 1:1 solution of PBS and Matrigel® (Corning). 10,000 mammospheres/animal were injected into the mammary fat pad of female, ovariectomized *FoxN1* nu/nu athymic nude mice (Harlan Sprague-Dawley, Madison, WI). E_2_ + control animals were also implanted with a 0.30 cm silastic capsule containing E_2_. Animals were fed by oral gavage with vehicle or 20 mg/kg of each individual PE (NG, RES or Q) 5 days per week for 8 weeks. Tumor area (L x W) was measured weekly. After 8 weeks, tumors were excised and measured for total volume and rate of tumor incidence was recorded for each treatment group.

### Statistical analysis

All experiments were conducted in triplicate at a minimum and repeated three independent times, with results reported as Mean ± Standard Deviation (S.D.). Comparisons between more than two groups were performed using ANOVA with a post-hoc Tukey’s test using GraphPad Prism 6 or 8 software. All graphs were designed using GraphPad Prism 6 or 8. The Kaplan–Meier curve for the in vivo analysis of TIC-potential was generated using GraphPad and statistical differences between the two experimental groups (SCBi vs. DAXXi) was calculated by the Log-rank, Mantel-Cox test. Linear regression analysis was performed on tumor growth studies to assess slopes of each tumor as each mouse was tagged with a number and tumor area was measured over an 11-week period. An unpaired, two-sided Student’s *t*-Test was performed on the slopes of each tumor growth curve to assess significance between two groups.

### Reporting summary

Further information on research design is available in the Nature Research Reporting Summary linked to this article.

## Supplementary information

Supplementary Information

Reporting Summary

## Data Availability

The raw and processed datasets generated during the current study, are publicly available in the figshare repository: 10.6084/m9.figshare.12601724^40^. Additional datasets supporting Supplementary Figs. 1–4, will be made available on reasonable request from the corresponding author, as described in the figshare data record above. Uncropped blots are publicly available as part of the supplementary information files.
