# Supplementary material for: DAXX-inducing phytoestrogens inhibit ER+ tumor initiating cells and delay tumor development
Source: NPJ Breast Cancer. 2020 Aug 14;6:37. doi: 10.1038/s41523-020-00178-5 (PMC7429502; doi:10.1038/s41523-020-00178-5)

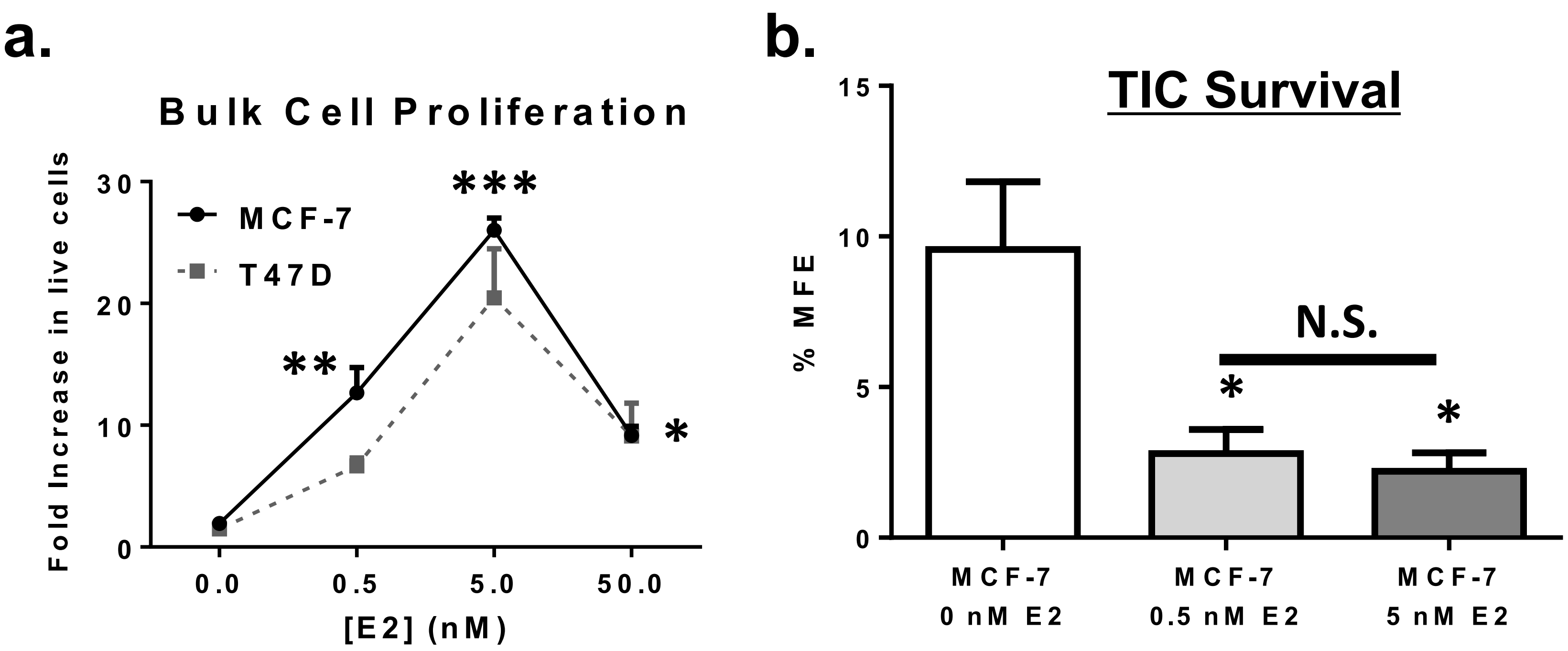

Supplementary Figure 1. Estradiol dose dependent responses of total bulk cell proliferation and TIC survival of ER+ breast cancer cells. **A.** MCF-7 and T47D cells were grown on 2 dimensional culture and with vehicle (0nM E_2) or increasing doses of E_2 for 7 days. Cells were removed from their plates with trypsin, the solution was quenched with growth media containing 0nM E2, and the pellet was re-suspended in growth medium containing 0nM E2. Cells were stained with trypan blue and the number of live cells was determined using an Invitrogen Countess Cell Counter. Fold increase in live cells were determined using the equation Fold increase in live cells= (Total live cells counted/100,000 cells originally plated). Graph shows mean +/- s.d. of 3 replicates. Statistical significance in Fold increase in live cells was calculated using a Student's *T*-Test, *= P< 0.05, **= P< 0.01, ***= P< 0.001. **B.** MCF-7 cells were grown on 2 dimensional culture and with vehicle (0nM E_2) or increasing doses of E_2 for 7 days. 50,000 cells from each group were plated into an ultra-low attachment plate containing methylcellulose mammosphere forming medium. After 7 days, mammospheres were imaged at 20X magnification, harvested, and %MFE calculated. Bar graphs show %MFE ± s.d. from three independent experiments. Statistical significance was calculated using a Student's *T*-Test. Symbols denote statistical significance: *= p< 0.01, NS= Not significant.

MCF-7 Cells

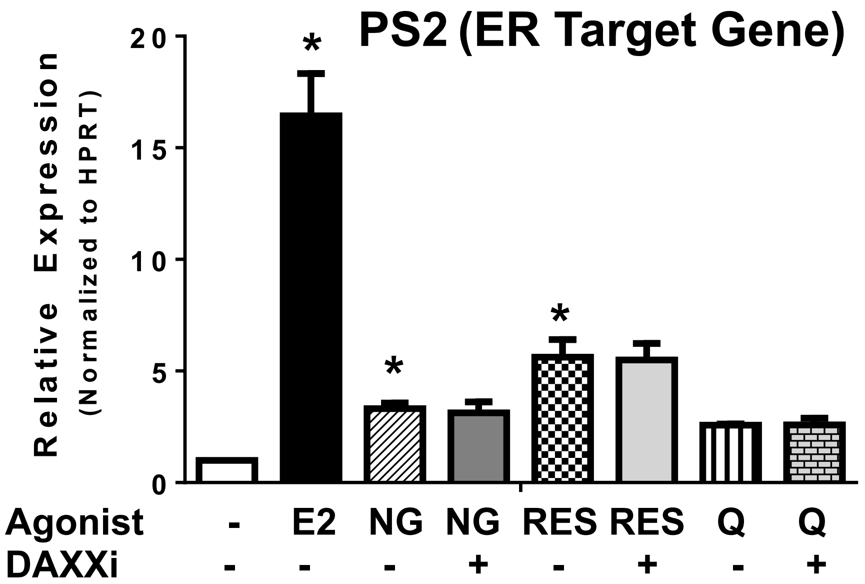

T47D Cells

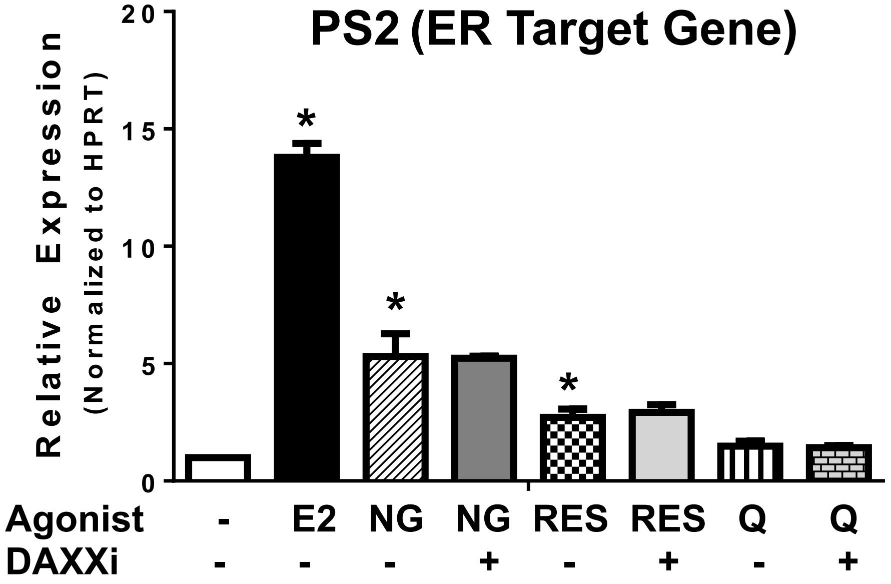

Supplementary Figure 2. Phytoestrogens modestly stimulate classical ER signaling. MCF-7 or T47D cells-used to generate Figure 3A at 3 days had their total RNA was isolated and reverse transcribed to cDNA. Real-time PCR was used to detect transcript levels of an ER target gene, PS2 (TFF1). Bar graphs show mean values ± S.D. of relative transcript expression normalized to HPRT and compared to SCBi + 0nM E_2 conditions from three independent experiments using the $2^{-\Delta\Delta Ct}$ calculation. Bar graphs show mean ± s.d. from three independent experiments. A One-way ANOVA was performed on ΔCt values after initial normalization to HPRT. Symbols denote statistical significance of p<0.01 between 5nM E_2, NG, RES, or Q vs vehicle (*) or controli vs DAXXi.

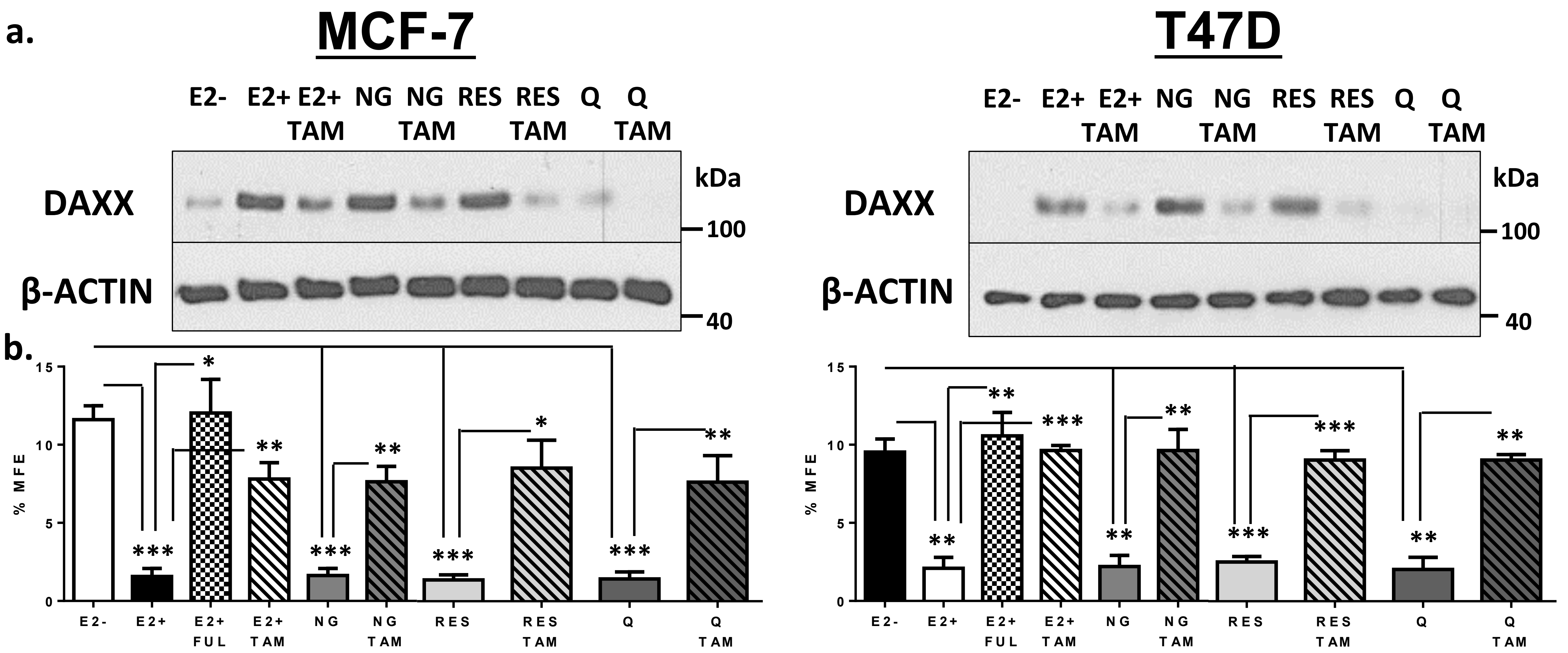

Supplementary Figure 3. Tamoxifen reverses PE-induced DAXX protein expression and is insufficient to restrict TIC-survival. MCF-7 and T47D cells were grown in 2 dimensional culture for 3 days under vehicle, E_2 (5nM) or PE (100nM) supplemented conditions in the presence or absence of 4-hydroxytamoxifen (TAM, 100nM). **a.** A subset of cells had their total protein isolated and DAXX proteins levels were determined by Western blotting. Images are representative of 3 replicates. **b.** A subset of 50,000 remaining cells used to generate Figure 2A were isolated and plated into an ultra-low attachment plate containing methylcellulose mammosphere forming medium. After 7 days, mammospheres were imaged at 20X magnification, harvested, and %MFE calculated. Bar graphs show mean %MFE ± s.d. from three independent experiments. Statistical significance was calculated using a One-way ANOVA with a Tukey post-hoc test for multiple comparisons. Symbols denote statistical significance between all groups compared to SCBi E_2-deprived (E2-) conditions as well as each other. *= P< 0.05, **=P< 0.01 ***= P< 0.001.

Supplementary Figure 4. MCF-7 cells were transfected with a control or DAXX siRNA for 48 hours. Transfected cells were injected into mammary fat pads of female, ovariectomized, *foxn1* nu/nu, athymic nude mice. Five mice received no estrogen capsule (E2-), 5 received an estrogen capsule (E2+), 5 were fed by oral gavage 20mg/Kg naringenin (NG), 20mg/Kg resveratrol (RES), or 20mg/Kg quercetin (Q). At 8 weeks, tumors were excised and images were taken. Ruler is in cm.

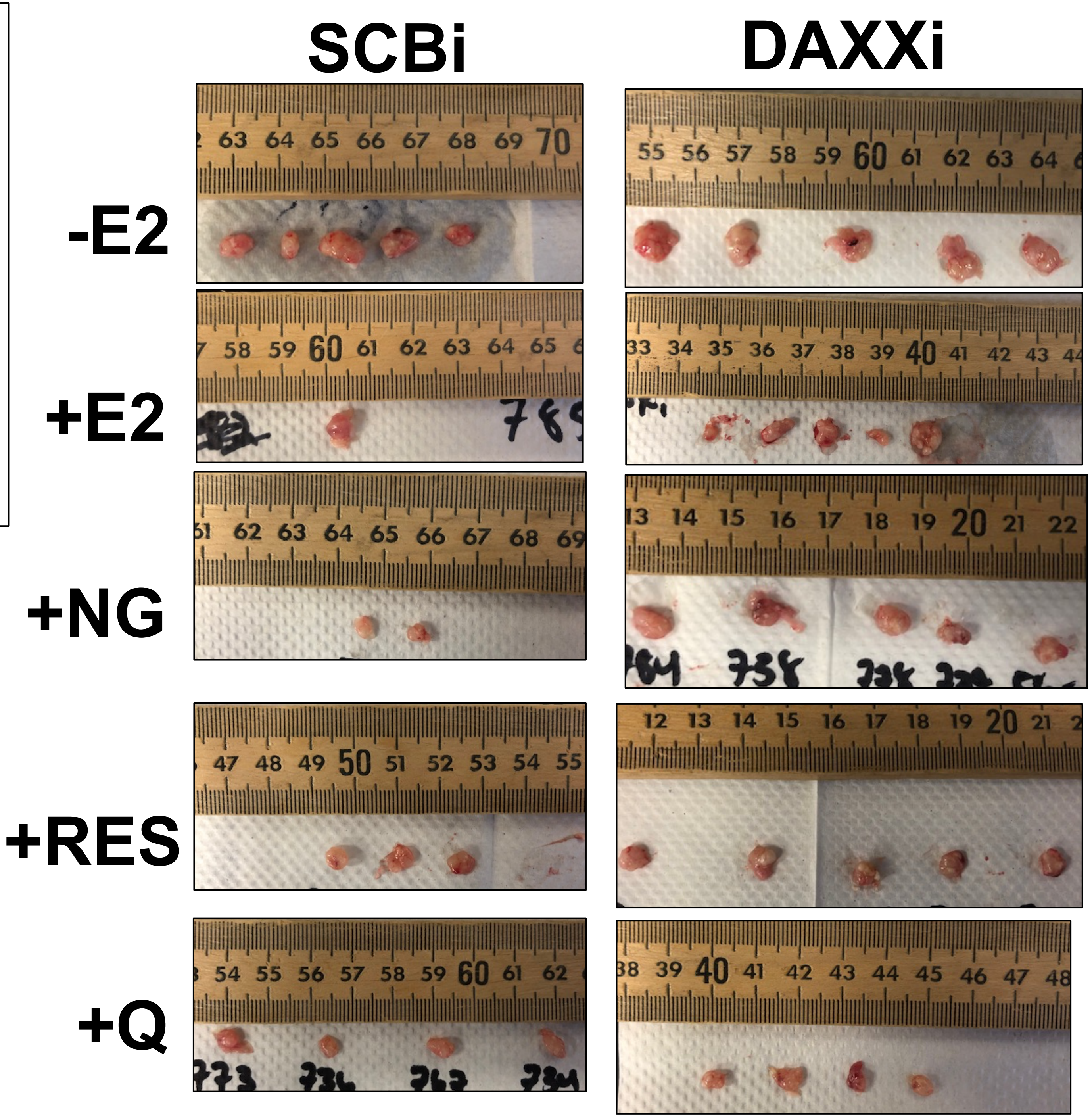

Supplementary Table 1: Primer Sequences for Real-time PCR

RT-PCR Primers	.
Primer Name	Sequence
HPRT Forward	GCCTATAGACTATCAGTTCCCTTTG
HPRT Reverse	TGTTTCACTCAATAGTGCTGTGG
PS2 Forward	GAGGCCCAGACAGAGACGTG
PS2 Reverse	CCCTGCAGAAGTGTCTAAAATTCA
HEY1 Forward	CCTGGGACTGCCATATTTTC
HEY1 Reverse	CCAGTTCAGTGGAGGTCGTT
HES1 Forward	CGGACATTCTGGAAATGACA
HES1 Reverse	CATTGATCTGGGTCATGCAG
SOX2 Forward	CACACTGCCCCTCTCAC
SOX2 Reverse	TCCATGCTGTTTCTTACTCTCC
OCT4 Forward	GGCAACCTGGAGAATTTGTTC
OCT4 Reserve	GTTACAGAACCACACTCGGAC
NANOG Forward	AGAGAATGAAATCTAAGAGGTGGC
NANOG Reverse	GTGGTAGGAAGAGTAAAGGCTG
NOTCH4 Forward	AACTCCTCCCCAGGAATCTG
NOTCH4 Reverse	CCTCCATCCAGCAGAGGTT

Figure 1B

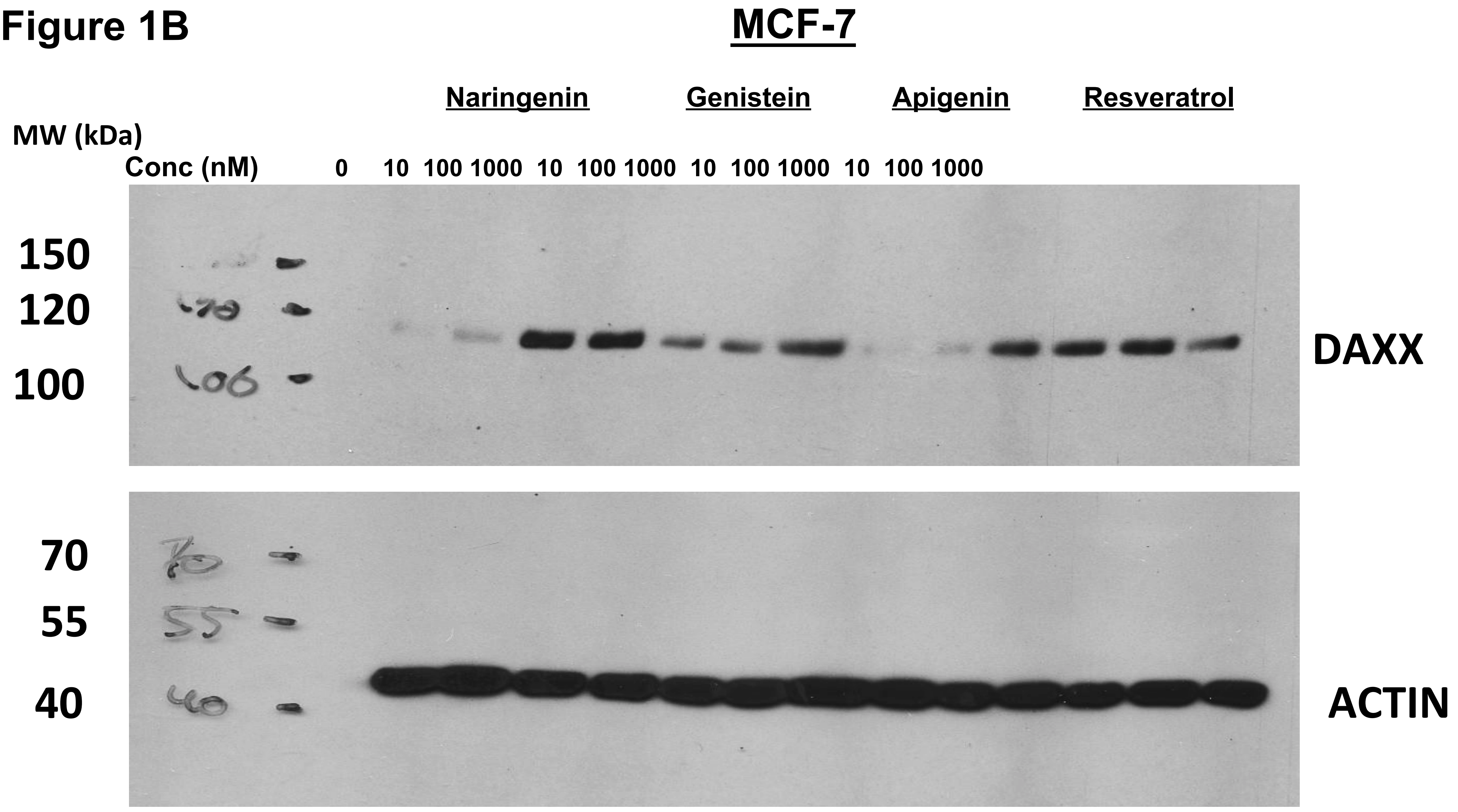
MCF-7
Naringenin
Genistein
Apigenin
Resveratrol
MW (kDa)
Conc (nM)
0
10 100 1000 10 100 1000 10 100 1000 10 100 1000
150
120
100
DAXX
70
55
40
ACTIN

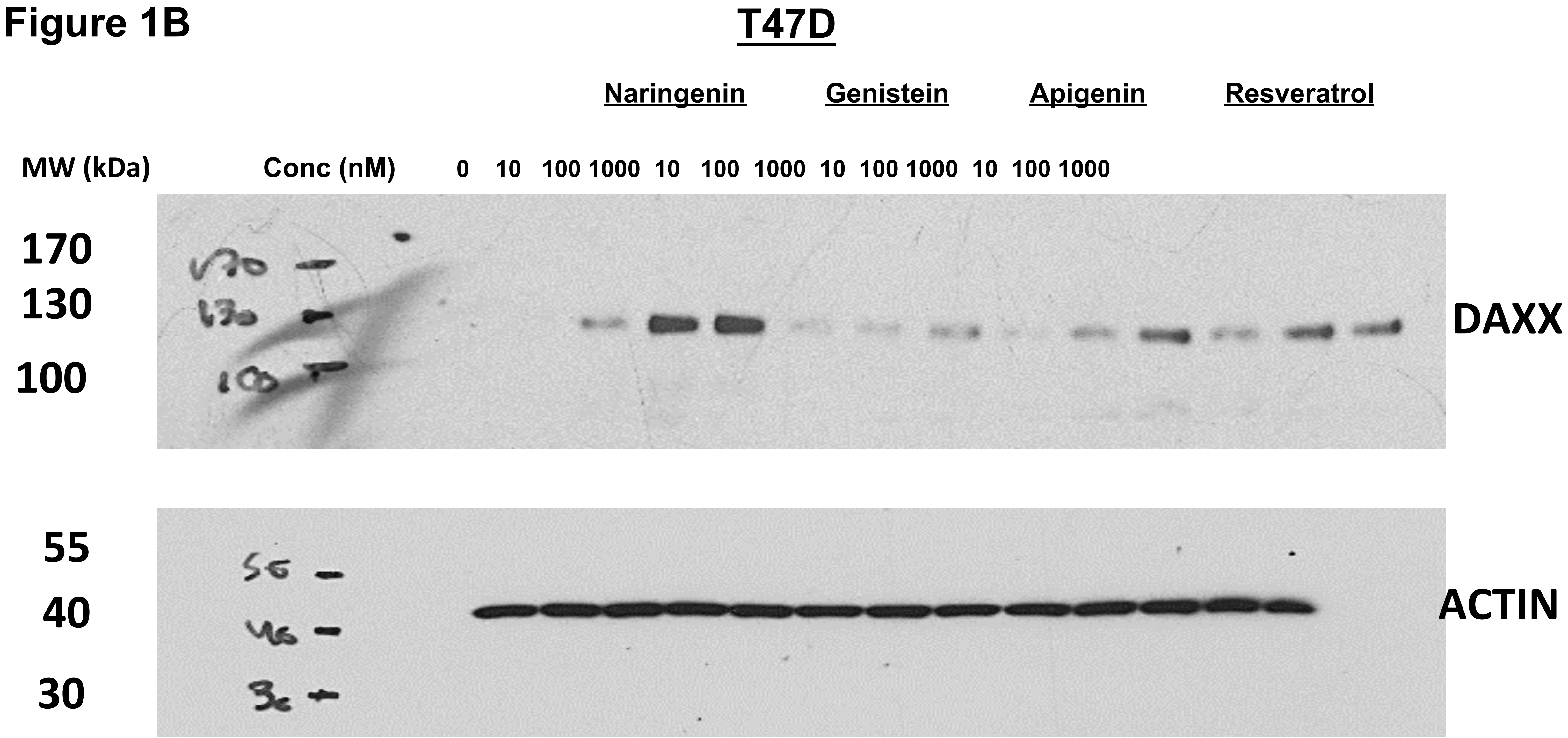
Figure 1B
T47D
Naringenin
Genistein
Apigenin
Resveratrol
MW (kDa)
Conc (nM)
0 10 100 1000 10 100 1000 10 100 1000 10 100 1000
170
130
100
DAXX
55
40
30
ACTIN

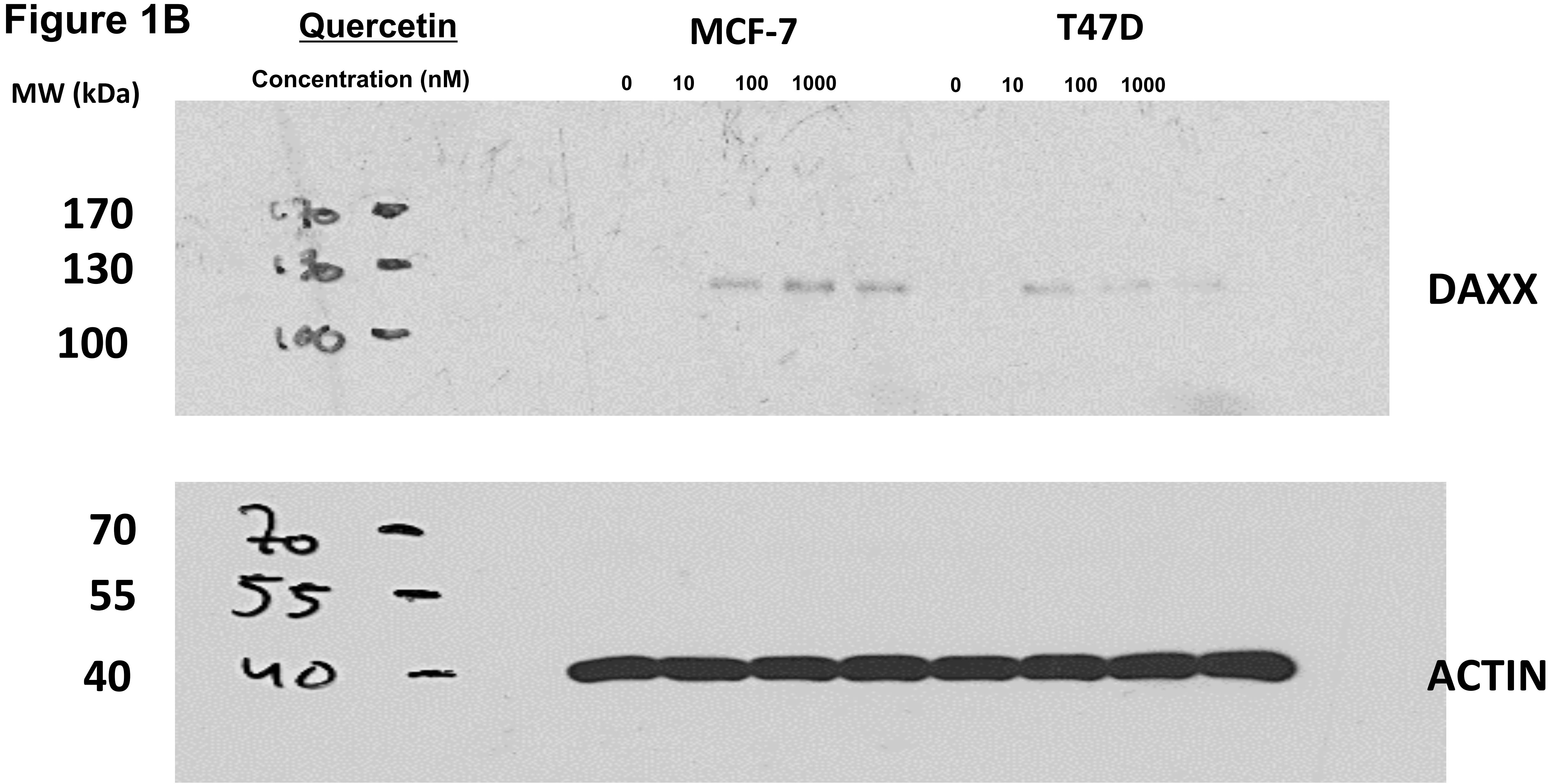
Figure 1B
Quercetin
Concentration (nM)
MCF-7
T47D
0 10 100 1000
0 10 100 1000
MW (kDa)
170
130
100
DAXX
70
55
40
ACTIN

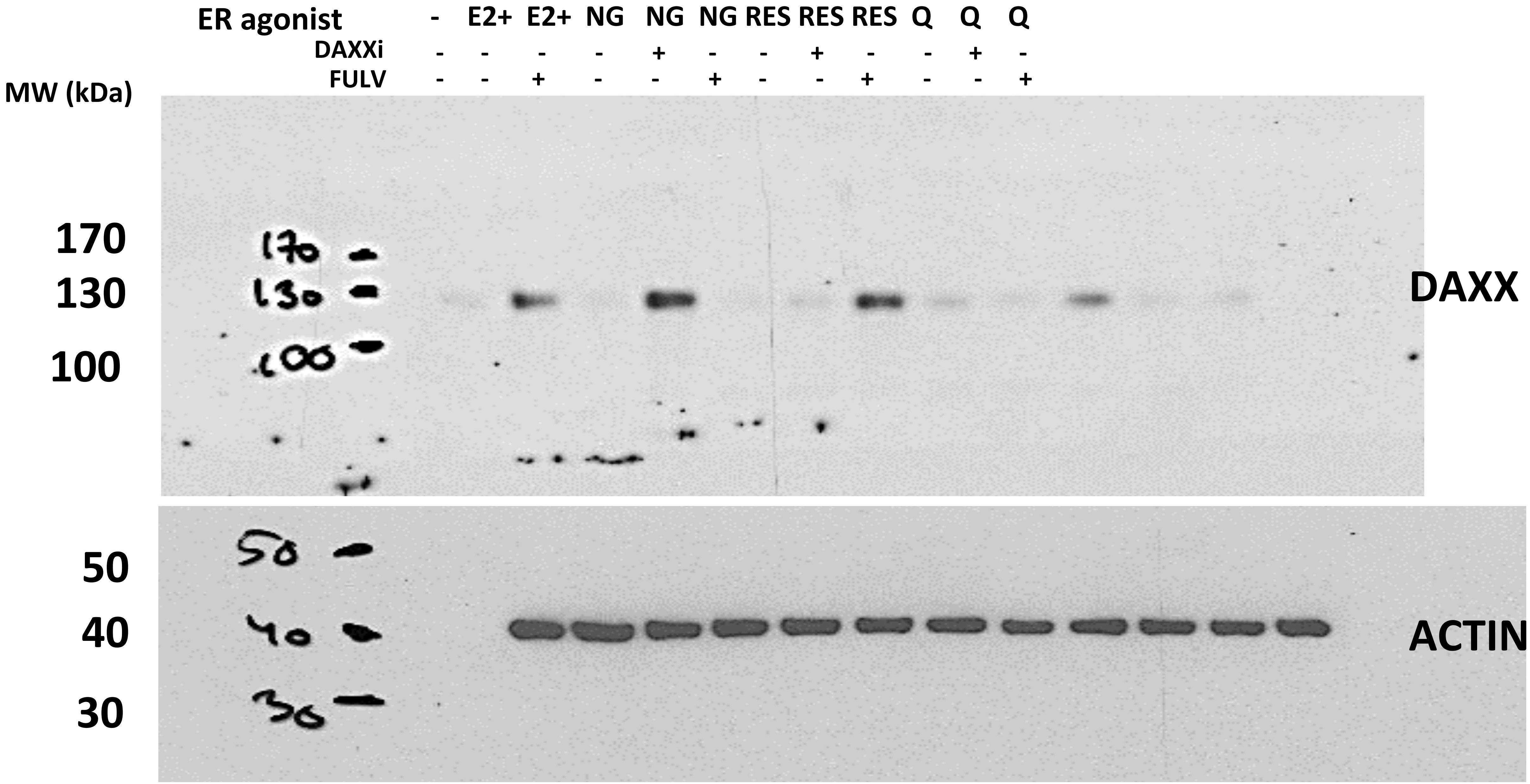

Figure 2A

MCF-7

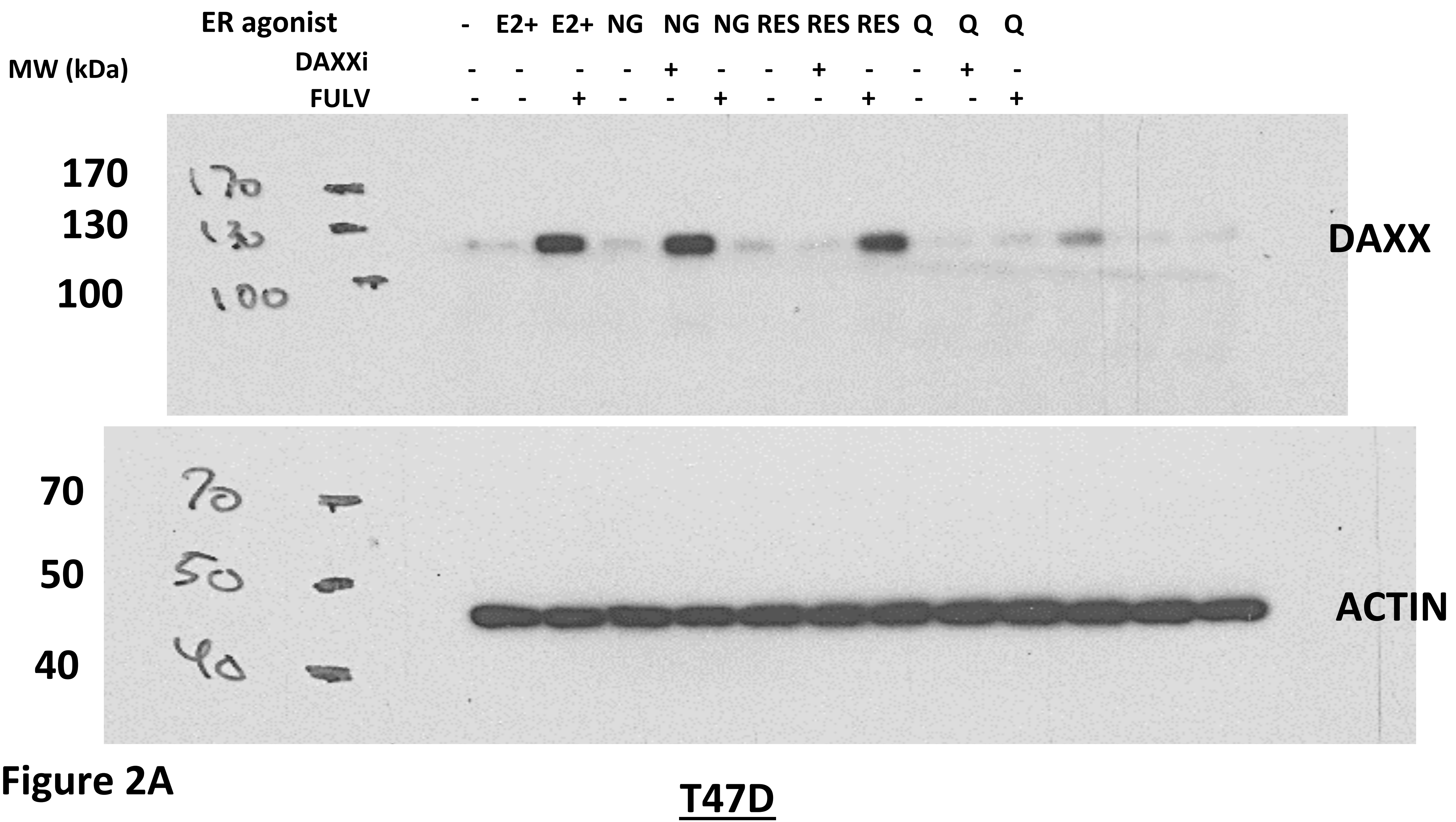
ER agonist - E2+ E2+ NG NG NG RES RES RES Q Q Q
MW (kDa)
DAXXi - - - - + - - + - - + -
FULV - - + - - + - - + - - +
170
130
100
DAXX
70
50
40
ACTIN
T47D
Figure 2A

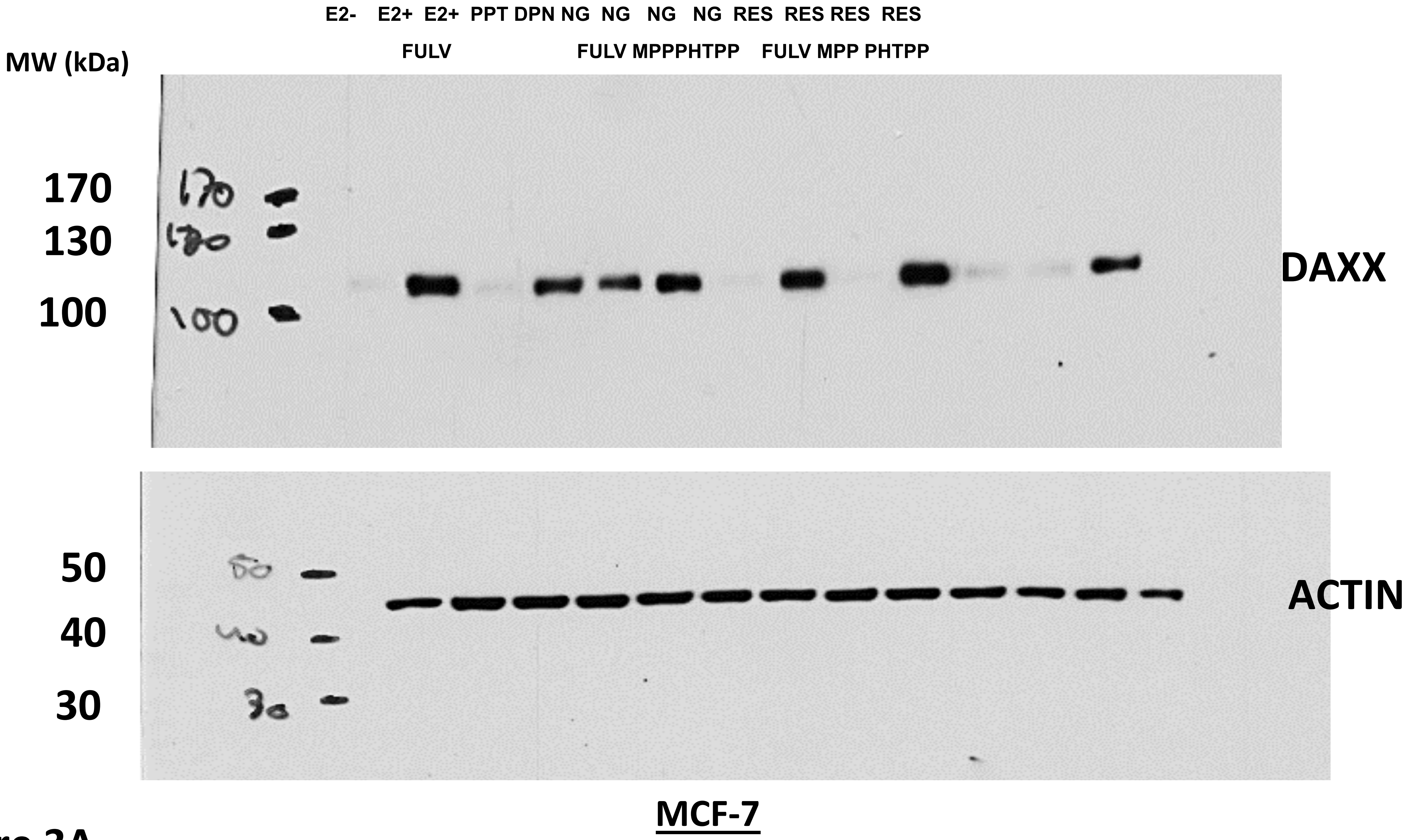
E2- E2+ E2+ PPT DPN NG NG NG NG RES RES RES RES
FULV FULV MPP PHTPP FULV MPP PHTPP
MW (kDa)
170
130
100
DAXX
50
40
30
ACTIN
MCF-7

Figure 3A

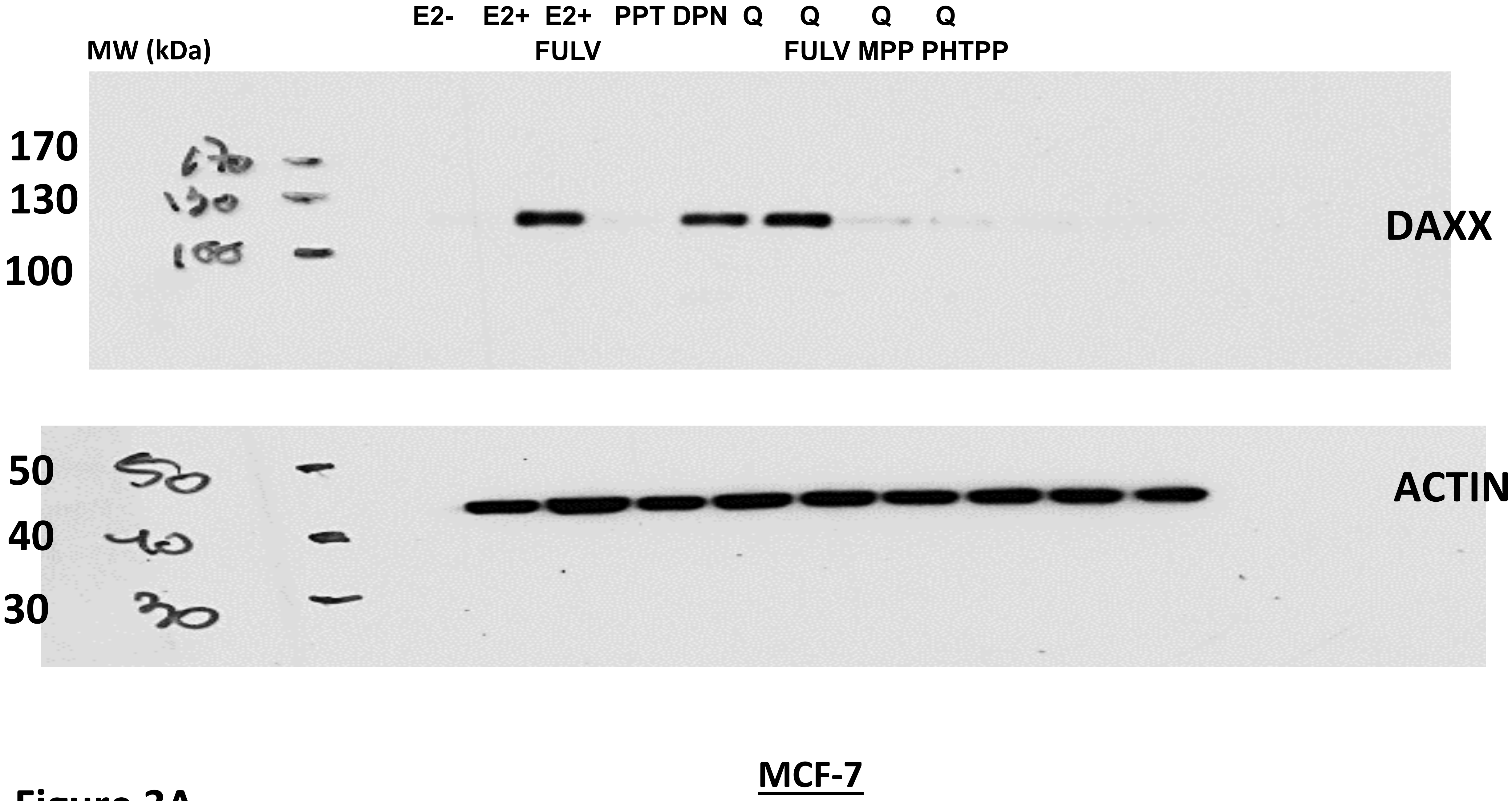
E2-
E2+
E2+ FULV
PPT
DPN
Q
Q FULV
Q MPP
Q PHTPP
MW (kDa)
170
130
100
DAXX
50
40
30
ACTIN
MCF-7

Figure 3A

Figure 3A

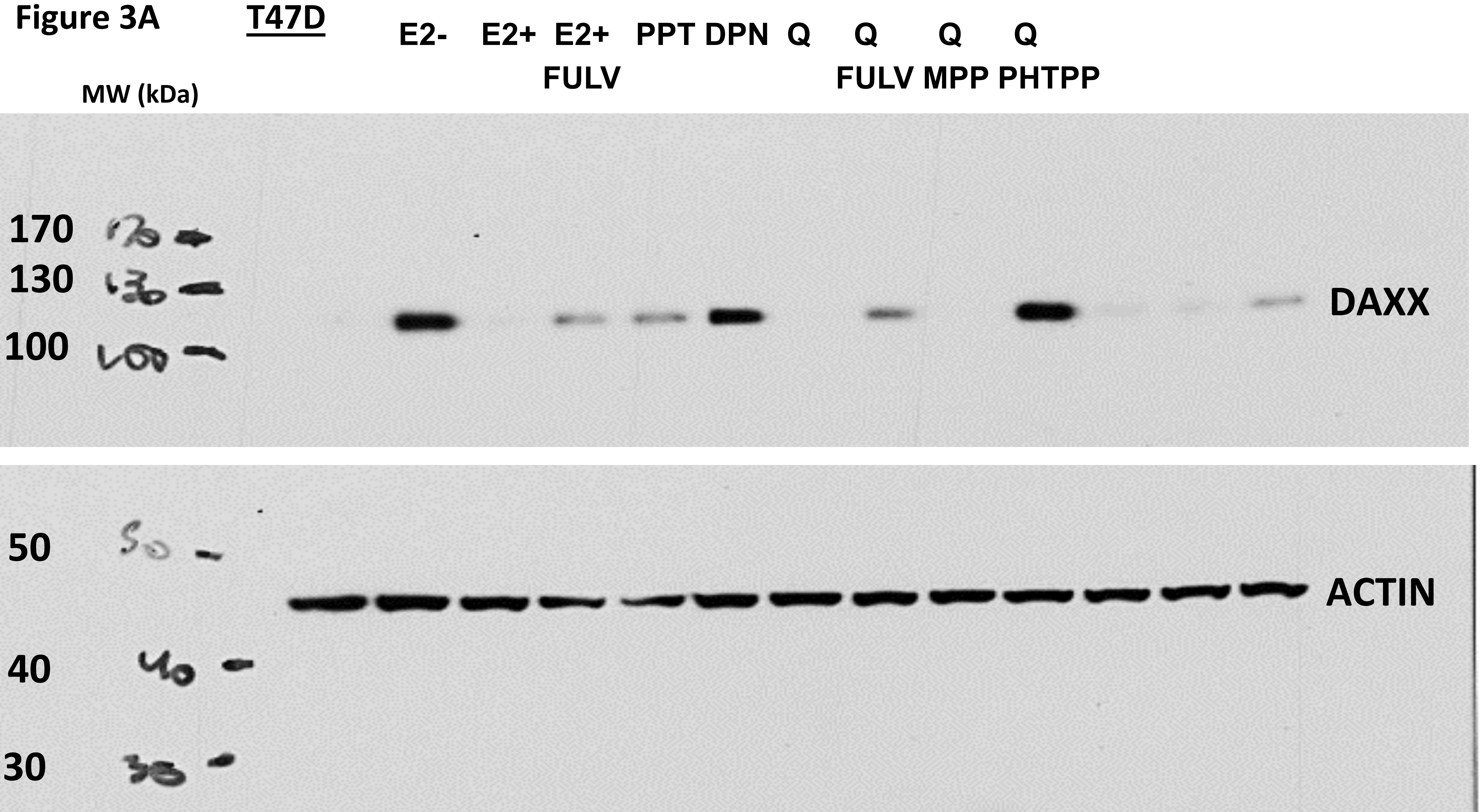

T47D
E2-
E2+
E2+ FULV
PPT
DPN
Q
Q FULV
Q MPP
Q PHTPP
MW (kDa)
170
130
100
DAXX
50
40
30
ACTIN

Figure 3A

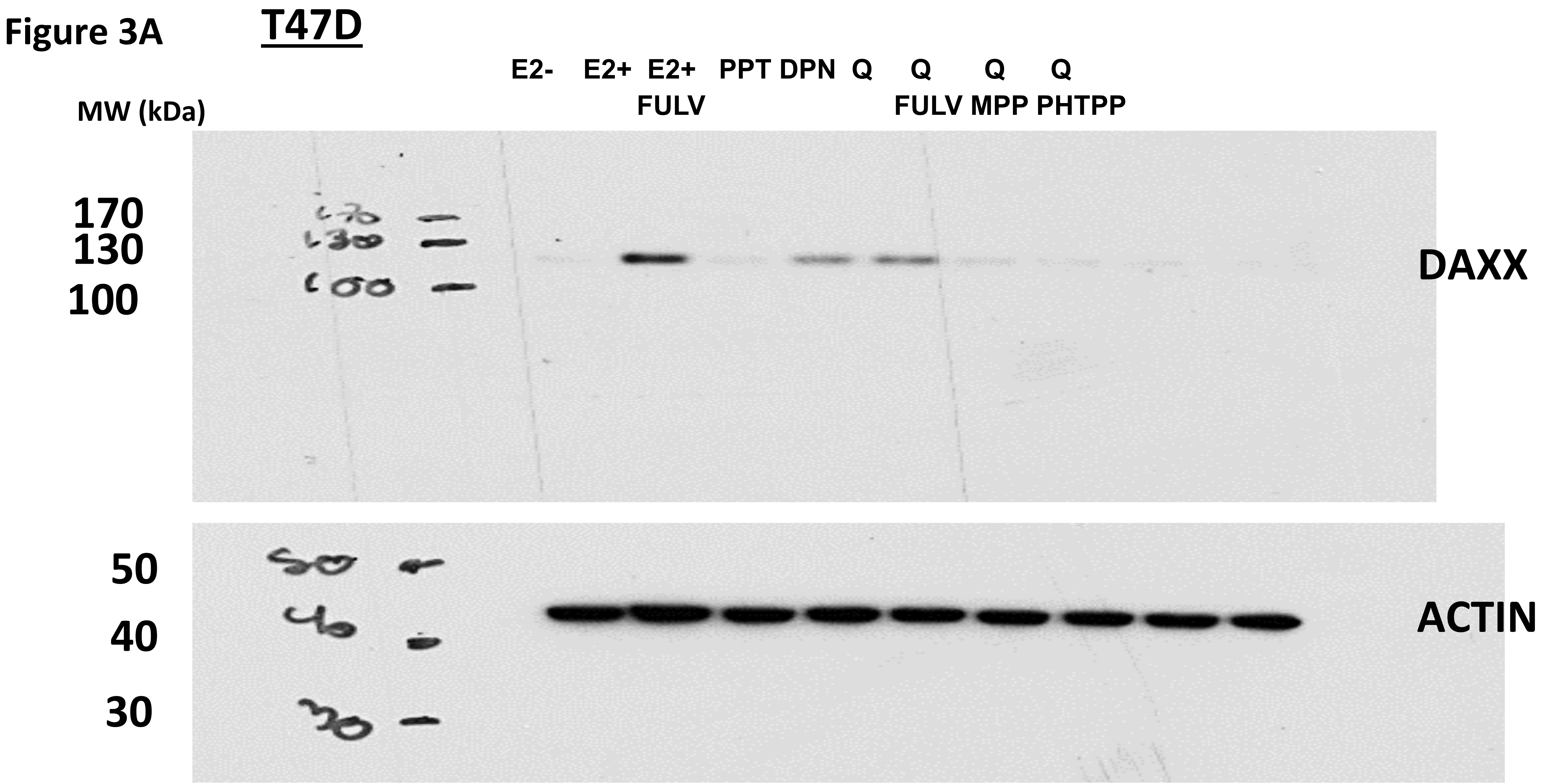
T47D
E2- E2+ E2+ PPT DPN Q Q Q Q
FULV FULV MPP PHTPP
MW (kDa)
170
130
100
DAXX
50
40
30
ACTIN

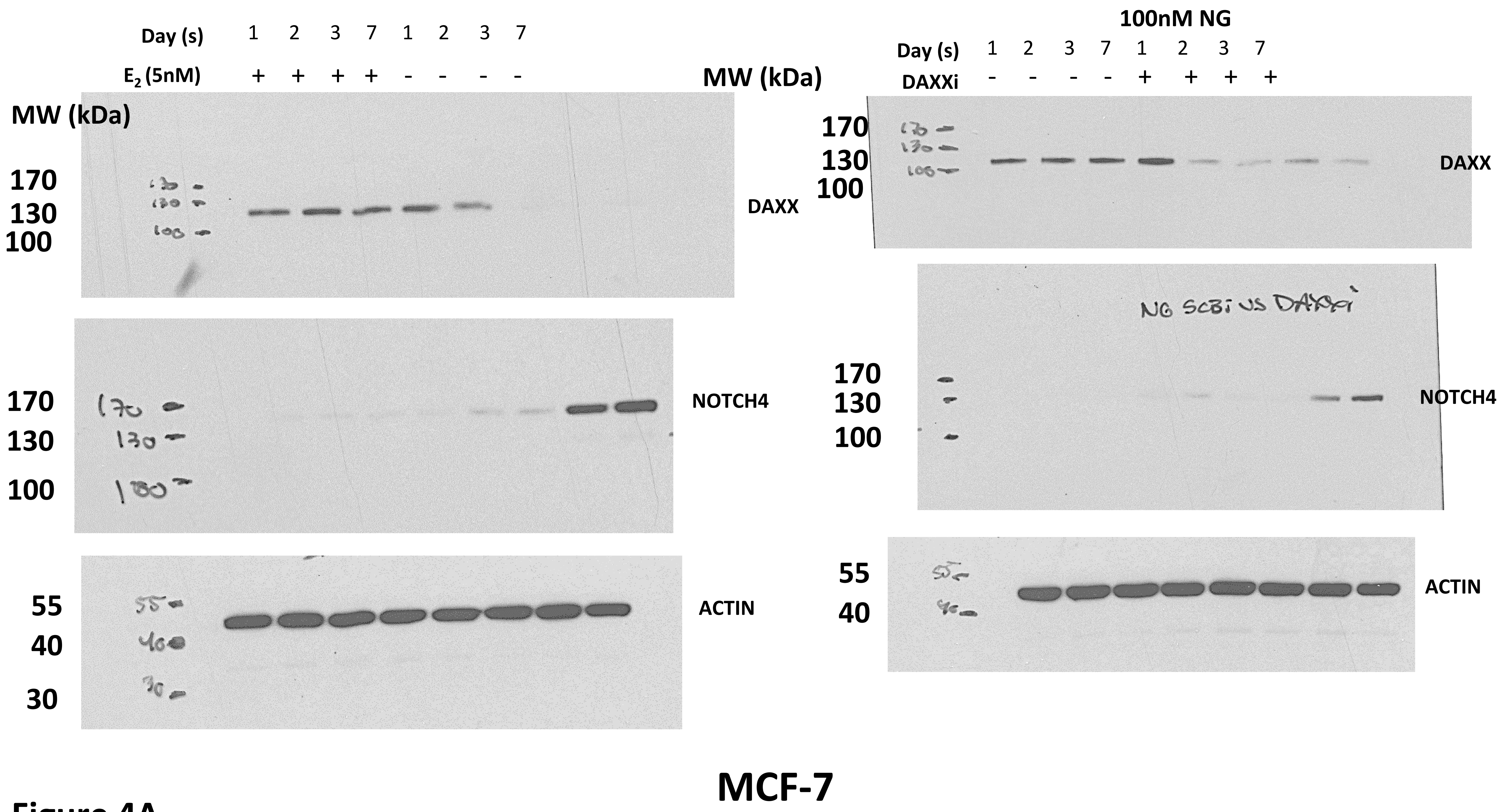
Day (s) 1 2 3 7 1 2 3 7
E2 (5nM) + + + + - - - -
MW (kDa)
170
130
100
DAXX
170
130
100
NOTCH4
55
40
30
ACTIN
100nM NG
Day (s) 1 2 3 7 1 2 3 7
DAXXi - - - - + + + +
MW (kDa)
170
130
100
DAXX
170
130
100
NOTCH4
55
40
ACTIN
MCF-7

Figure 4A

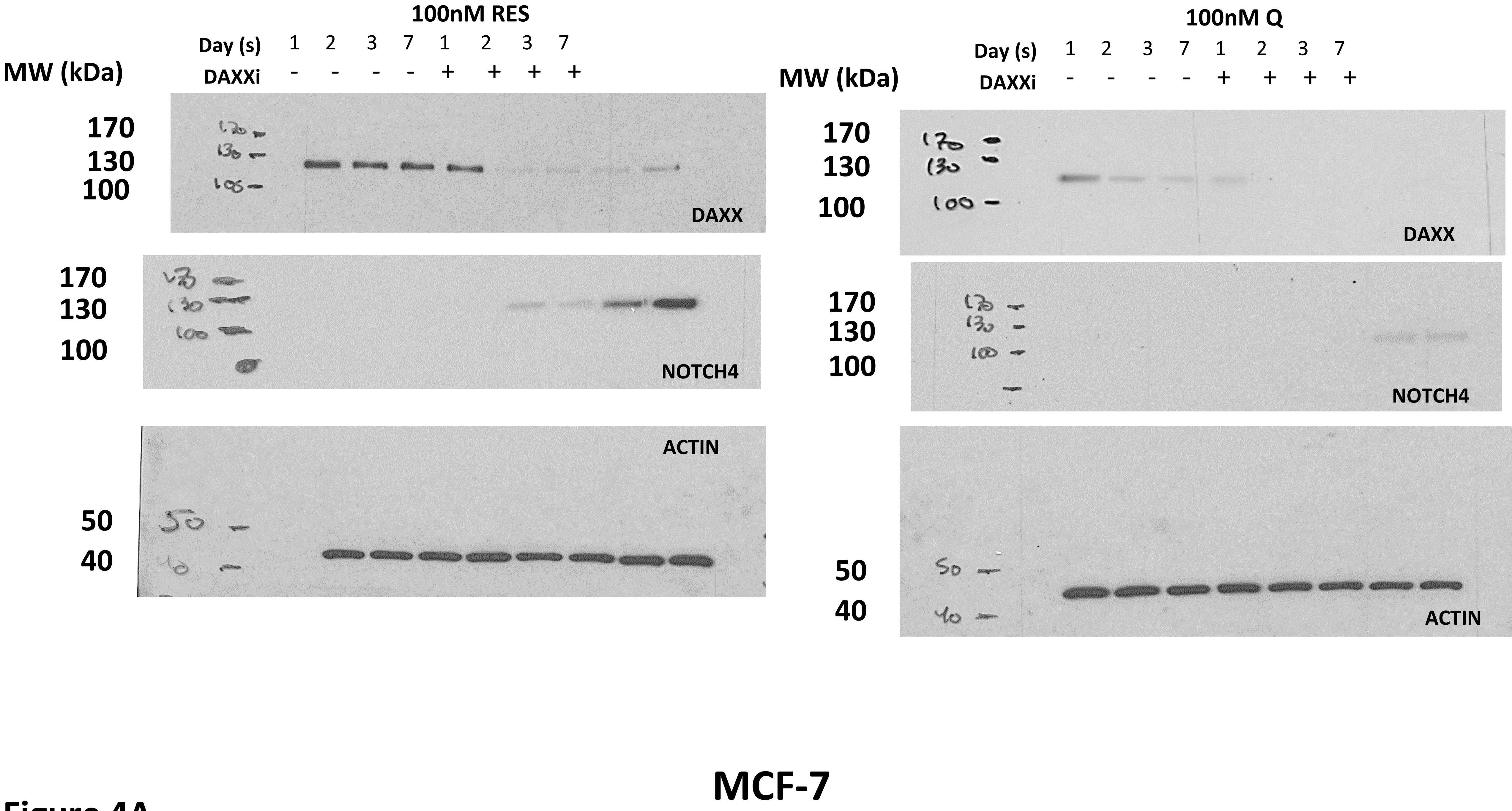
100nM RES
Day (s) 1 2 3 7 1 2 3 7
MW (kDa)
DAXXi - - - - + + + +
170
130
100
DAXX
170
130
100
NOTCH4
ACTIN
50
40
100nM Q
Day (s) 1 2 3 7 1 2 3 7
MW (kDa)
DAXXi - - - - + + + +
170
130
100
DAXX
170
130
100
NOTCH4
50
40
ACTIN
MCF-7

Figure 4A

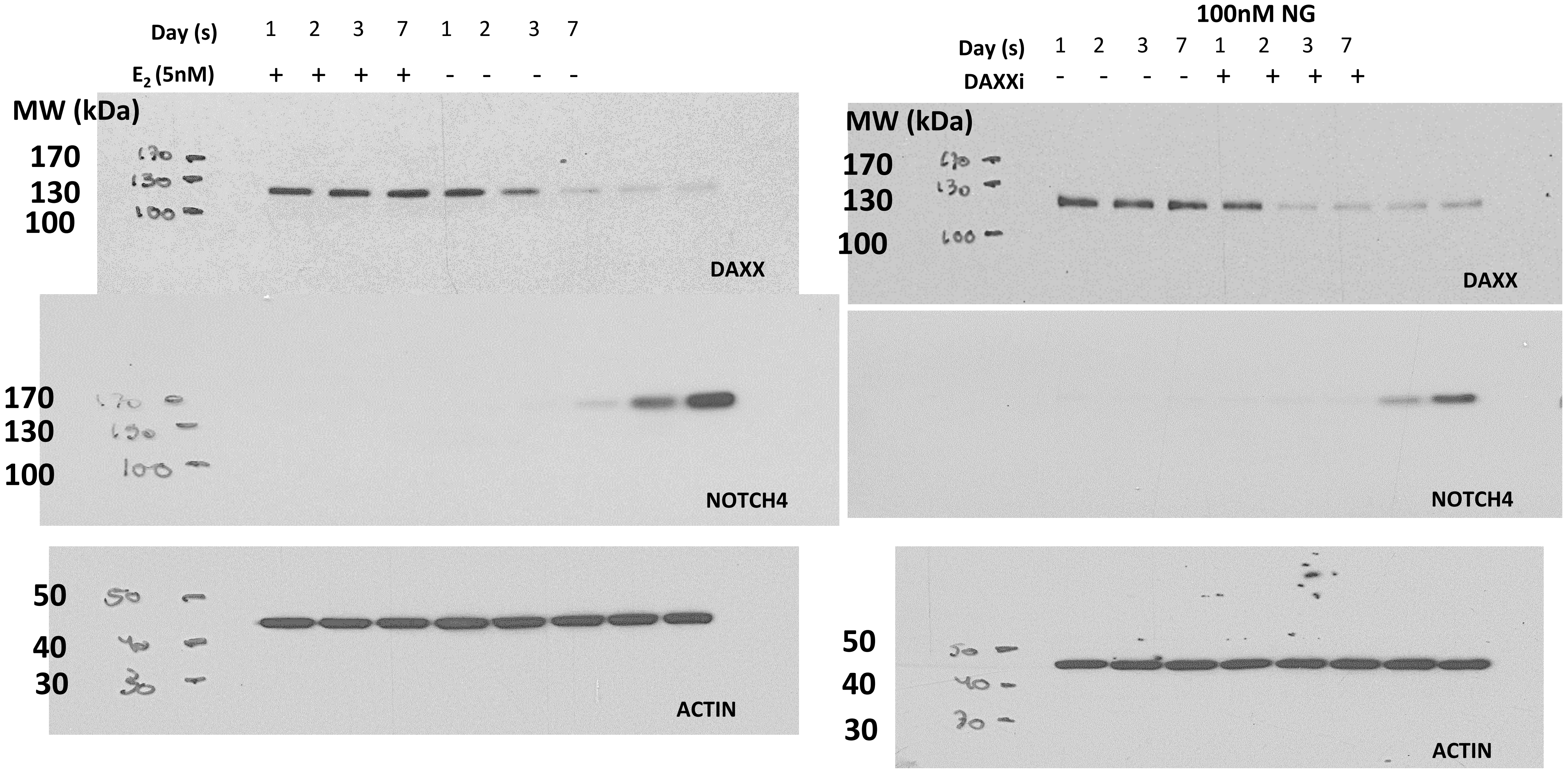

T47D

Figure 4B

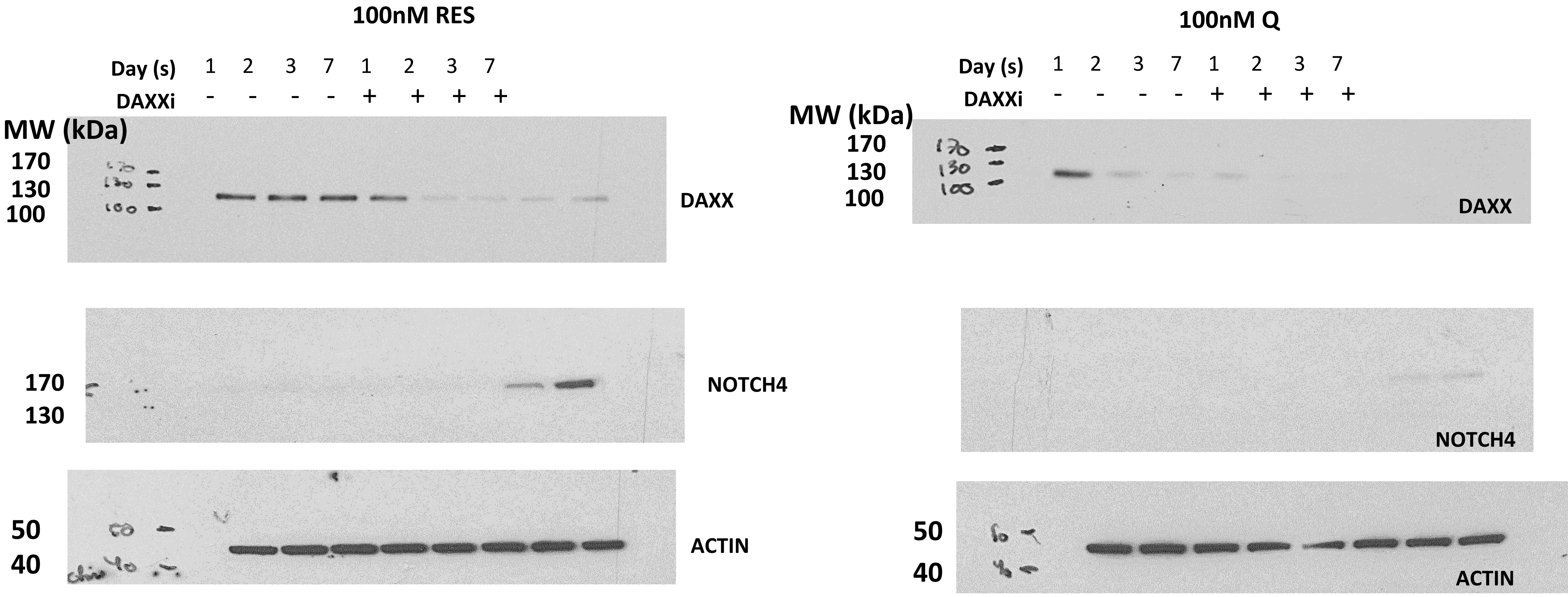

T47D

Figure 4B

Figure 6A

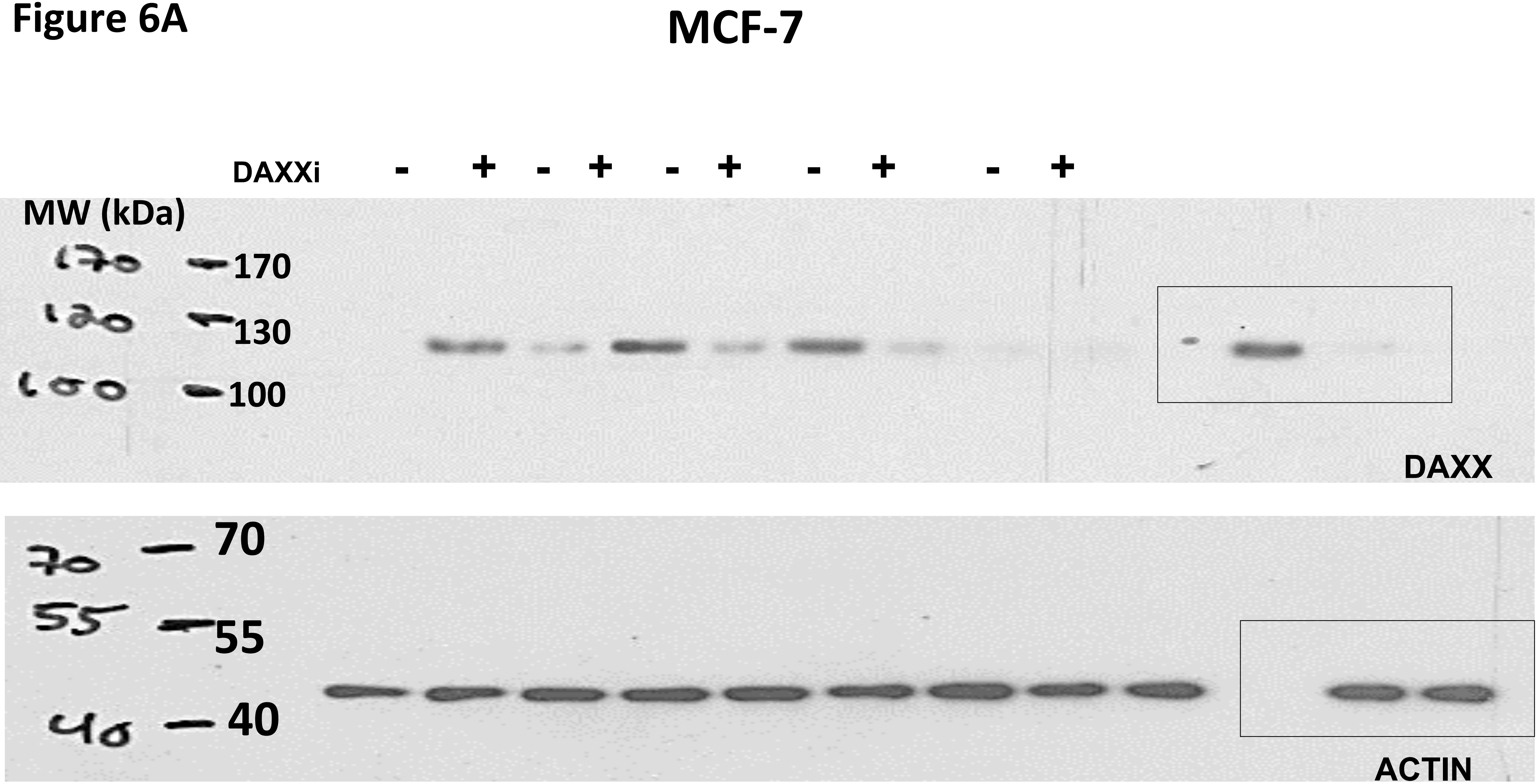

Figure 7A

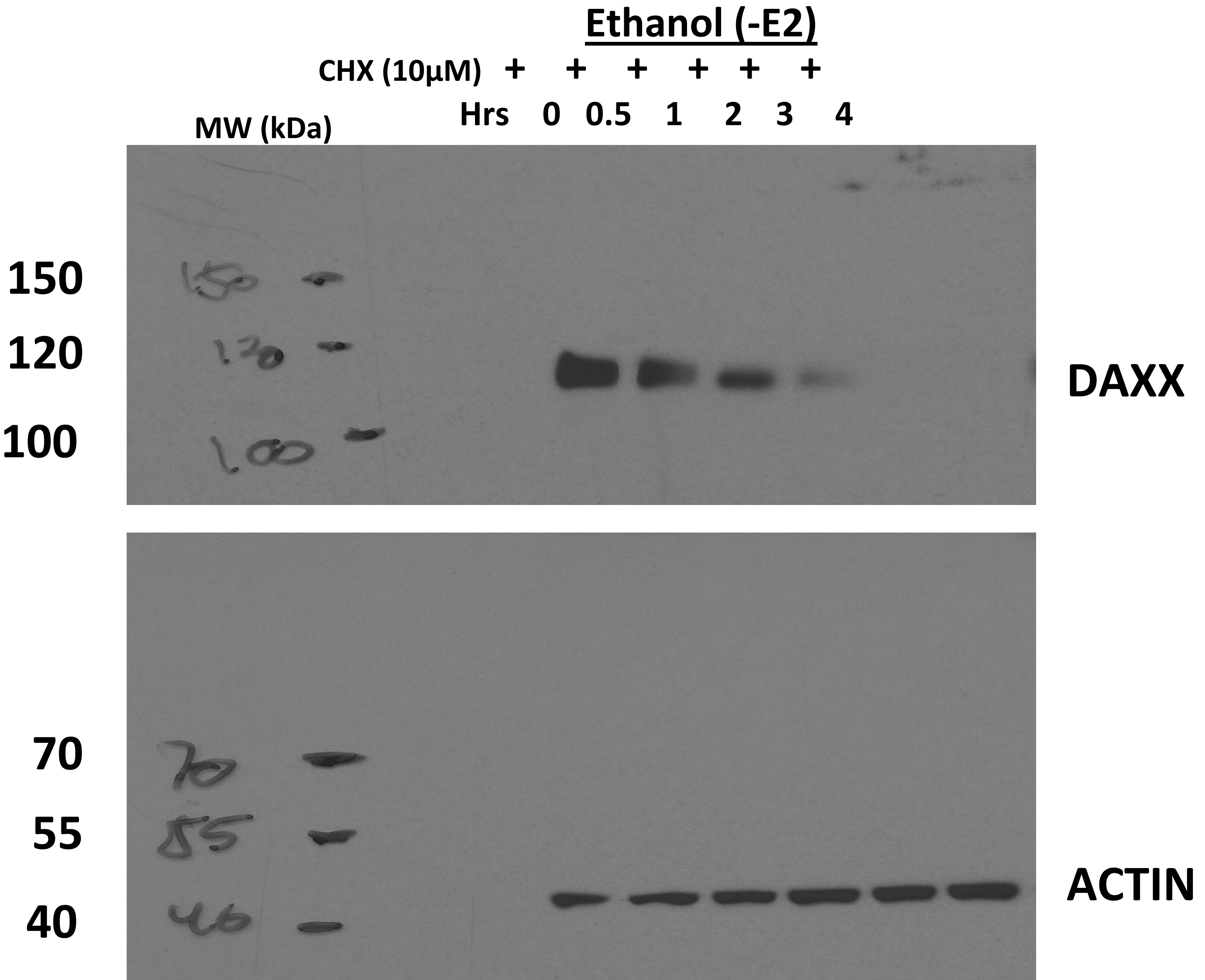

Ethanol (-E2)
CHX (10µM) + + + + + +
Hrs 0 0.5 1 2 3 4
MW (kDa)
150
120
100
DAXX
70
55
40
ACTIN

Figure 7A

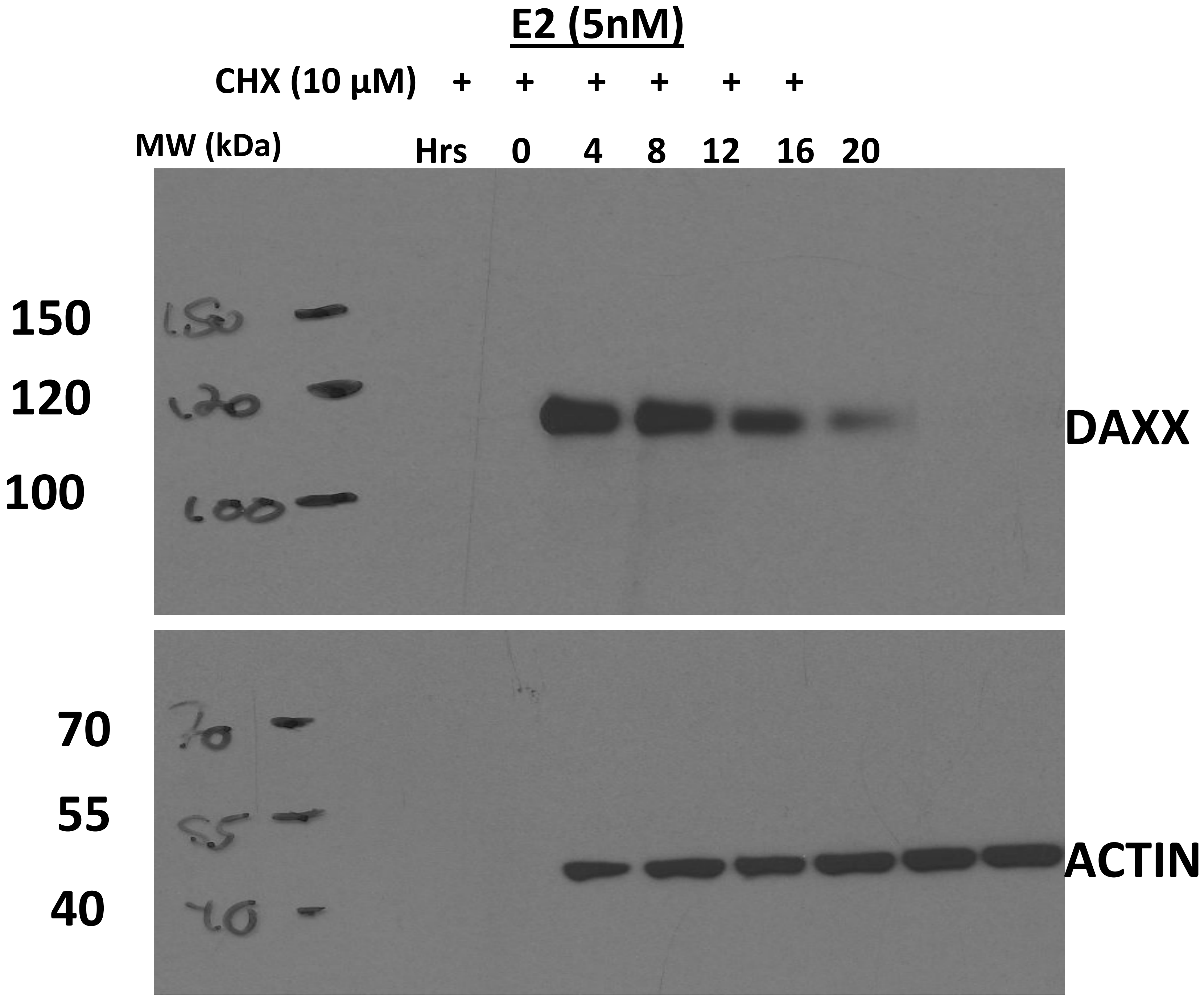
E2 (5nM)
CHX (10 μM) + + + + + +
MW (kDa)
Hrs 0 4 8 12 16 20
150
120
100
DAXX
70
55
40
ACTIN

Figure 7A

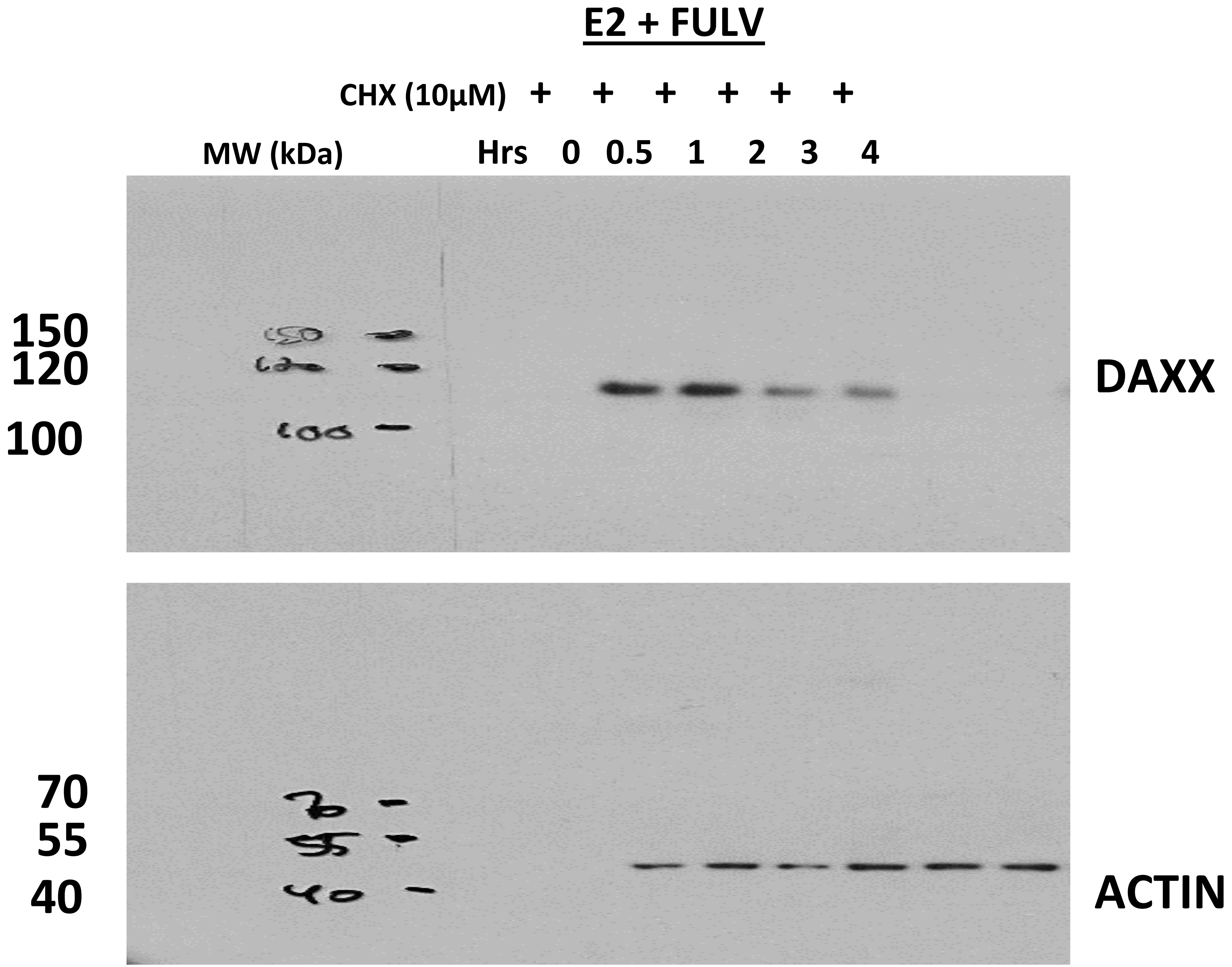
E2 + FULV
CHX (10μM) + + + + + +
MW (kDa)
Hrs 0 0.5 1 2 3 4
150
120
100
DAXX
70
55
40
ACTIN

Figure 7A

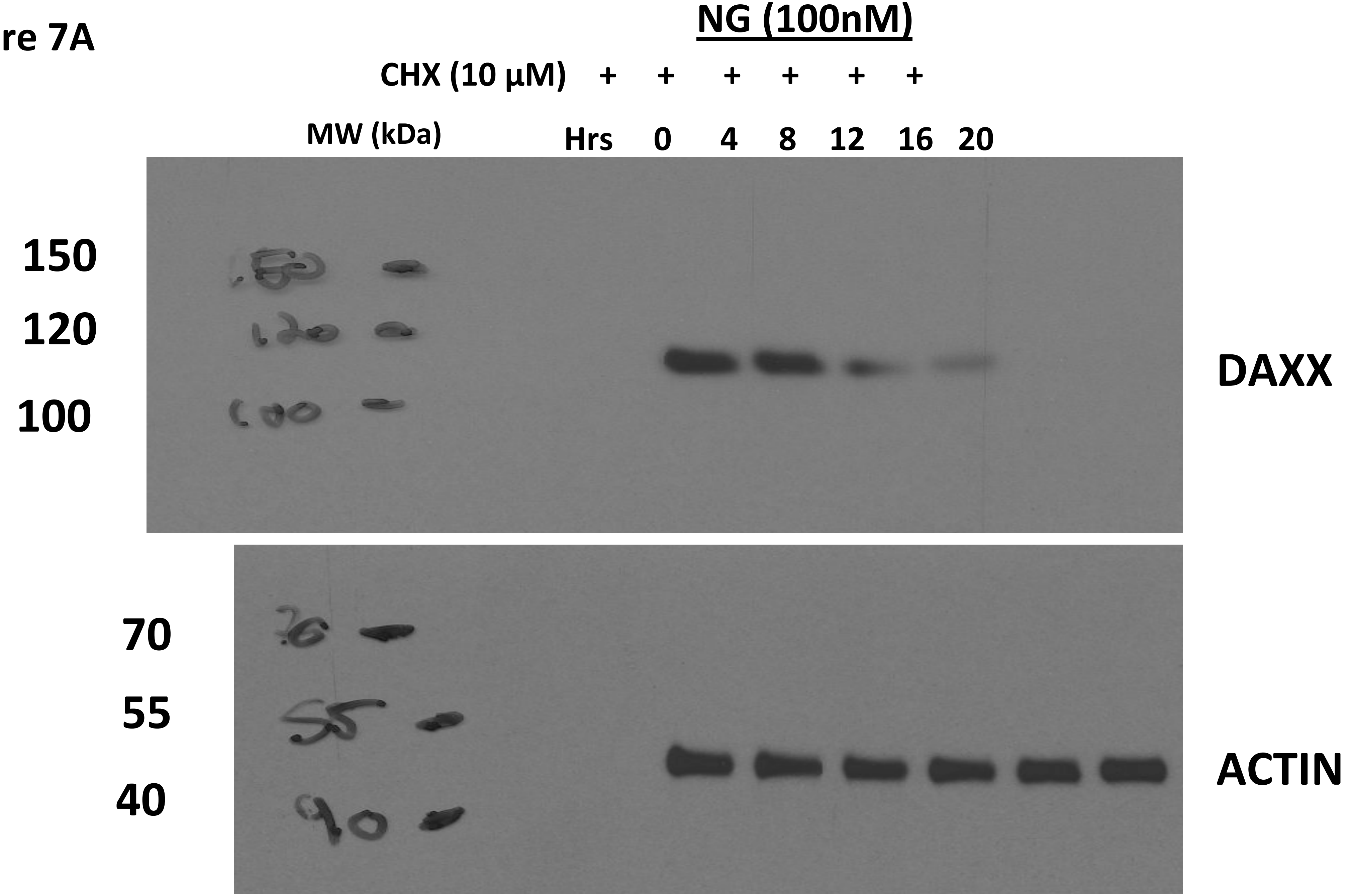
NG (100nM)
CHX (10 µM) + + + + + +
MW (kDa)
Hrs 0 4 8 12 16 20
150
120
100
DAXX
70
55
40
ACTIN

Figure 7A

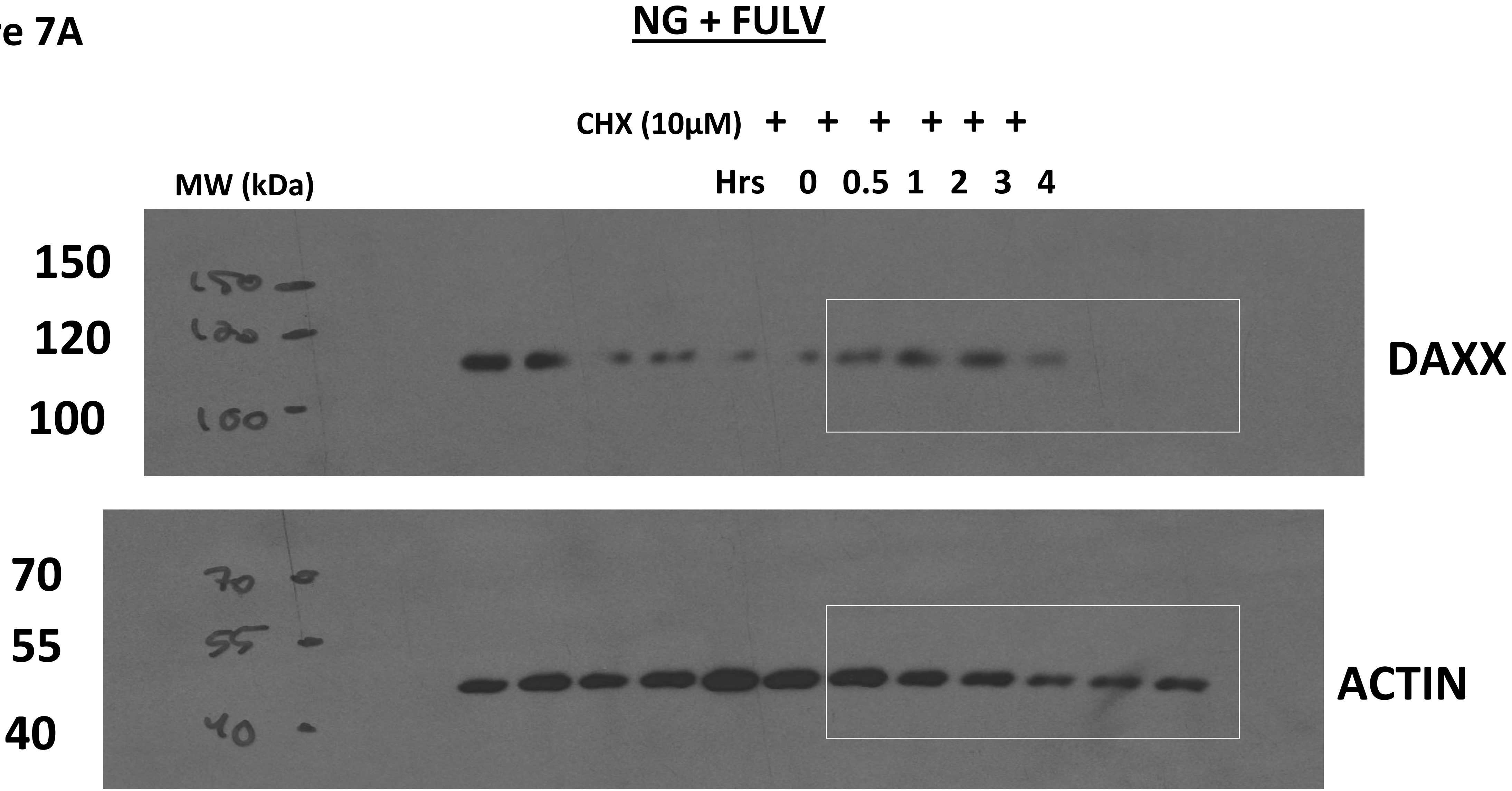
NG + FULV
CHX (10μM) + + + + + +
Hrs 0 0.5 1 2 3 4
MW (kDa)
150
120
100
DAXX
70
55
40
ACTIN

Figure 9B

ER+ PDX BCM 5097

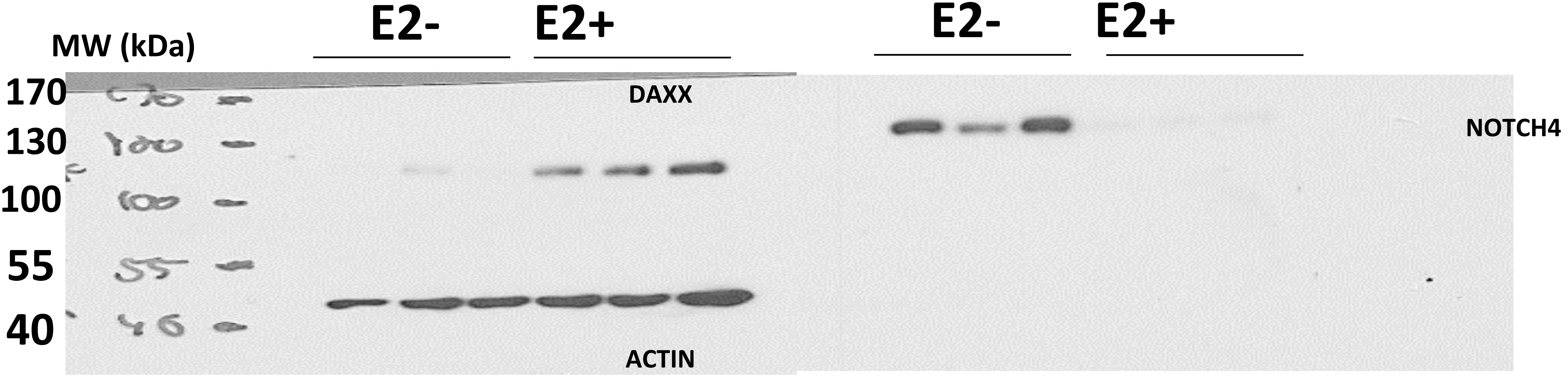

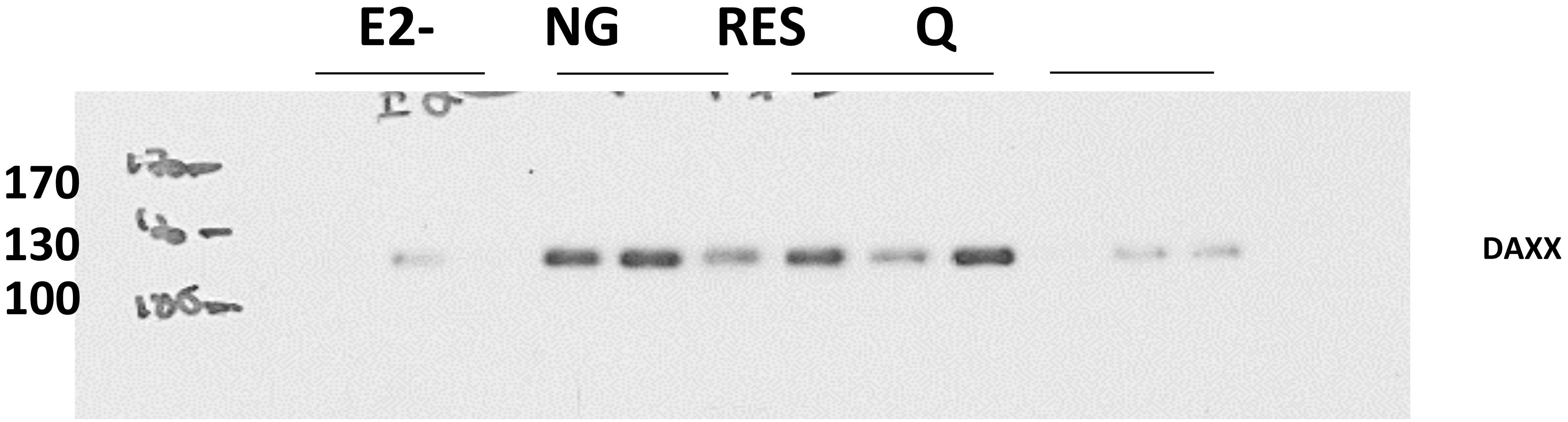

Figure 9B

ER+ PDX BCM 5097

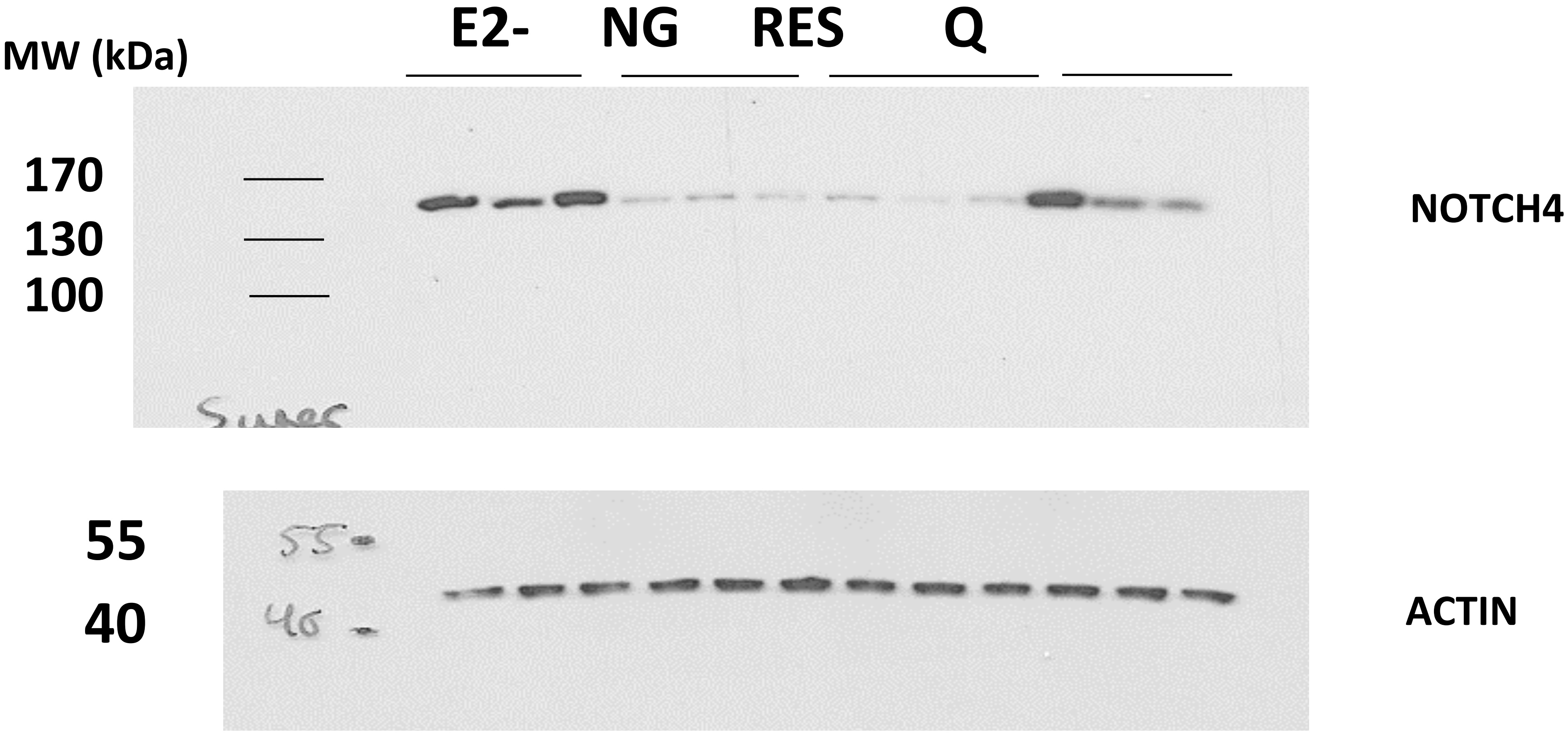

Supplement: Supplementary file 1 — Supplementary Information [file 41523_2020_178_MOESM1_ESM.pdf]
