# Supplementary material for: DAXX-inducing phytoestrogens inhibit ER+ tumor initiating cells and delay tumor development
Source: NPJ Breast Cancer. 2020 Aug 14;6:37. doi: 10.1038/s41523-020-00178-5 (PMC7429502; doi:10.1038/s41523-020-00178-5)
Supplement: Supplementary file 2 — Reporting Summary [file 41523_2020_178_MOESM2_ESM.pdf]

nature research

Corresponding author(s): Clodia Osipo

Last updated by author(s): 05/29/2020

Reporting Summary

Nature Research wishes to improve the reproducibility of the work that we publish. This form provides structure for consistency and transparency in reporting. For further information on Nature Research policies, see our Editorial Policies and the Editorial Policy Checklist.

Statistics

For all statistical analyses, confirm that the following items are present in the figure legend, table legend, main text, or Methods section.

n/a	Confirmed	
☐	☒	The exact sample size (n) for each experimental group/condition, given as a discrete number and unit of measurement
☐	☒	A statement on whether measurements were taken from distinct samples or whether the same sample was measured repeatedly
☐	☒	The statistical test(s) used AND whether they are one- or two-sided<br>*Only common tests should be described solely by name; describe more complex techniques in the Methods section.*
☒	☐	A description of all covariates tested
☐	☒	A description of any assumptions or corrections, such as tests of normality and adjustment for multiple comparisons
☐	☒	A full description of the statistical parameters including central tendency (e.g. means) or other basic estimates (e.g. regression coefficient) AND variation (e.g. standard deviation) or associated estimates of uncertainty (e.g. confidence intervals)
☐	☒	For null hypothesis testing, the test statistic (e.g. F, t, r) with confidence intervals, effect sizes, degrees of freedom and P value noted<br>*Give P values as exact values whenever suitable.*
☒	☐	For Bayesian analysis, information on the choice of priors and Markov chain Monte Carlo settings
☒	☐	For hierarchical and complex designs, identification of the appropriate level for tests and full reporting of outcomes
☒	☐	Estimates of effect sizes (e.g. Cohen's d, Pearson's r), indicating how they were calculated

Our web collection on statistics for biologists contains articles on many of the points above.

Software and code

Policy information about availability of computer code

Data collection	Graph Pad Prism 6, Microsoft Excel 2016
Data analysis	Graph Pad Prism 6, Microsoft Excel 2016

For manuscripts utilizing custom algorithms or software that are central to the research but not yet described in published literature, software must be made available to editors and reviewers. We strongly encourage code deposition in a community repository (e.g. GitHub). See the Nature Research guidelines for submitting code & software for further information.

Data

Policy information about availability of data

All manuscripts must include a data availability statement. This statement should provide the following information, where applicable:

- Accession codes, unique identifiers, or web links for publicly available datasets
- A list of figures that have associated raw data
- A description of any restrictions on data availability

The authors declare that [the/all other] data supporting the fndings of this study are available within the paper [and its supplementary information files]. Any other data not included are available upon request.

Field-specific reporting

Please select the one below that is the best fit for your research. If you are not sure, read the appropriate sections before making your selection.

☒ Life sciences ☐ Behavioural & social sciences ☐ Ecological, evolutionary & environmental sciences

For a reference copy of the document with all sections, see nature.com/documents/nr-reporting-summary-flat.pdf

Life sciences study design

All studies must disclose on these points even when the disclosure is negative.

Sample size	Sample size for in vitro cell culture studies are based on experience and previous published reports. Sample size for animal studies were calculated using Power Analysis based on previous experience and published reports.
Data exclusions	No data were excluded
Replication	All attempts at replication were reproducible with similar findings.
Randomization	During animal studies, randomization was conducted based on mean tumor area (l x w) to maintain similar tumor area means for the treatment groups.
Blinding	All animal studies were conducted by a single individual and performed to the best of our knowledge in an unbiased manner. Animals were tagged with a number and tumor area per mouse was measured to the best of our knowledge in a blinded and unbiased manner.

Reporting for specific materials, systems and methods

We require information from authors about some types of materials, experimental systems and methods used in many studies. Here, indicate whether each material, system or method listed is relevant to your study. If you are not sure if a list item applies to your research, read the appropriate section before selecting a response.

Materials & experimental systems

n/a	Involved in the study	
☐	☒	Antibodies
☐	☒	Eukaryotic cell lines
☒	☐	Palaeontology and archaeology
☐	☒	Animals and other organisms
☒	☐	Human research participants
☒	☐	Clinical data
☒	☐	Dual use research of concern

Methods

n/a	Involved in the study	
☒	☐	ChIP-seq
☒	☐	Flow cytometry
☒	☐	MRI-based neuroimaging

Antibodies

Antibodies used	DAXX (Cell Signaling clone 25C12, rabbit anti-human, Catalog number 4533); Beta-Actin (Sigma Aldrich clone Ac-15, mouse anti-human, cat # A5441); Notch4 (Santa Cruz Biotechnology, clone A-12, monoclonal, cat# sc-393893)
Validation	For both DAXX and Notch4 antibodies, siRNA mediated knockdown confirmed specificity of detected proteins. For Actin, molecular weight was used to identify protein of interest.

Eukaryotic cell lines

Policy information about cell lines

Cell line source(s)	American Type Culture Collection
Authentication	All cell lines used in the study were authenticated in 2019 using STR allele profiling by ATCC
Mycoplasma contamination	All cell lines were tested for mycoplasm and tested negative.
Commonly misidentified lines (See ICLAC register)	NA

Animals and other organisms

Policy information about studies involving animals; ARRIVE guidelines recommended for reporting animal research

Laboratory animals	Female Foxn1 nu/nu athymic nude mice were used to generate cell line tumor xenografts. NSG female mice were used to implant and passage human breast PDX tumors.
Wild animals	NA
Field-collected samples	NA
Ethics oversight	Loyola University Chicago's Institutional Animal Care and Use Committee (IACUC).

Note that full information on the approval of the study protocol must also be provided in the manuscript.
